# A Moment Versus a Lifetime: Patterns of Loneliness and Perceived Causes in People's Lived Experiences

**DOI:** 10.1111/nyas.70082

**Published:** 2025-10-03

**Authors:** Luzia Cassis Heu

**Affiliations:** ^1^ Department of Interdisciplinary Social Sciences Utrecht University Utrecht the Netherlands

**Keywords:** attachment, chronic loneliness, hypervigilance, loneliness, social sensitivity

## Abstract

For effective loneliness interventions, we need a better understanding of why some loneliness experiences persist (often labeled chronic loneliness), while most loneliness experiences remain transient. To provide starting points for future research on causes of chronic loneliness, interview data from adults ages 19–45 years from India, Egypt, Turkey, Israel, Bulgaria, and Austria were reanalyzed. Because of little scientific consensus on the exact definition of chronic versus transient loneliness, different temporal patterns of loneliness were first distinguished in the data. Instead of two, four types emerged: Transient loneliness typically lasted some hours to 2 years; recurrent loneliness recurred every couple of weeks or months; prolonged loneliness lasted for multiple years; and chronic loneliness usually had its onset in childhood or adolescence and persisted for most people's lives. Perceived causes for loneliness were compared across those four temporal patterns, with findings showing that transient or prolonged loneliness was typically attributed to concrete external situations, but chronic loneliness was explained more by unfulfilling family relationships in childhood, perceptions that one does not fit in with societal norms, or high relationship expectations. Both recurrent and chronic loneliness were often attributed to sensitivity, rumination, overgeneralizations in relationships, or discomfort with oneself (e.g., low self‐acceptance).

## Graphical abstract

Qualitative data were reanalyzed to explore how the chronic loneliness that people do not recover from differs from transient loneliness in lived experiences, and to explore self‐perceived causes for different loneliness patterns. Not only two, but multiple patterns of loneliness were observed. Different from other loneliness patterns, chronic loneliness was often attributed to unfulfilling family relationships in childhood or not fitting in with social norms.

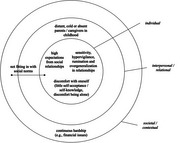

## 1 Introduction

Loneliness is uncomfortable and associated with multiple mental and physical health problems [1, 2]. Nevertheless, most people experience loneliness at some point in their lives and recover from it themselves [3]. Also, loneliness has been suggested to be an evolutionarily developed signal to reconnect with others, as social isolation has been a threat to survival throughout most of human history [4]. This suggests that not all loneliness experiences require intervention. Instead, interventions may, for instance, need to target the loneliness that people do not manage to recover from themselves. In the research literature, this is typically discussed as *chronic* loneliness—relatively long‐lasting or life‐long loneliness [5]. Such loneliness is not only unpleasant for a longer time than transient loneliness, it may also have a more continuous negative impact on health, and has, accordingly, been found to more strongly predict adverse health outcomes [6, 7].

While a categorization of loneliness experiences as either transient or chronic may help distinguish those who require treatment from those recover relatively quickly on their own, there is, thus far, little consensus on how to clearly differentiate between the two. A qualitative examination of loneliness patterns may help better understand what characterizes each type in practice and could ground future theorizing—or a decision about cut‐off points—in lived experiences. Additionally, it may provide practitioners—such as GPs or psychotherapists—with examples of how different types of loneliness manifest in the way people talk about their loneliness experiences, enabling more timely and appropriate interventions. This paper presents a reanalysis of 53 interviews about loneliness experiences from people ages 19–45 years from India, Egypt, Bulgaria, Israel, Austria, and Turkey [8, 9].

It also includes a first exploration of risk factors for chronic loneliness. We know little about why some people develop chronic loneliness while others recover from common loneliness‐inducing situations, such as separation or bereavement. Such knowledge is important to ensure interventions target the underlying causes of loneliness from which people do not recover, rather than addressing all forms or durations of loneliness indiscriminately.

### 1.1 Transient Versus Chronic Loneliness

Loneliness has been defined as the unpleasant feeling resulting from a perceived discrepancy between the relationships individuals have and those that they desire [10]. People may feel lonely not because they have poorer social networks compared to those who do not report loneliness, but because their relationships fail to meet their *expectations*. From an evolutionary psychological perspective, loneliness is an adaptive reaction to social disconnection and isolation, encouraging people to reconnect with others [4]. Just as hunger or pain, it signals the need for action to avoid harm. This suggests that loneliness is usually a transient state because it motivates behavior that erases the feeling. However, like pain, loneliness can become chronic. Loneliness experiences have, accordingly, been categorized based on their duration [5]. Short‐lived loneliness experiences are often labeled “transient loneliness”; because such experiences tend to also be bound to specific external situations, such as a break‐up or bereavement, they are often referred to as “situational loneliness.” By contrast, “chronic” loneliness typically describes longer‐lasting or life‐long loneliness.

Despite this conceptual distinction, there is little consensus on a precise cut‐off point between transient and chronic loneliness. Initially, chronic loneliness was defined to last for at least 2 years [11]. Existing research tends to operationalize chronic loneliness as a certain frequency or level of loneliness being reported at multiple consecutive measurement points [6, 7, 12, 13, 14]. For instance, in a study among Spanish adults, participants were categorized as chronically lonely if they reported loneliness at least sometimes at two measurement points separated by 3 years [7]. In a study among US American participants ages 50+ years, people were categorized as chronically lonely if they reported having felt lonely much of the time in the previous week, assessed at three time points, separated by 1 year [14]. People who reported feeling lonely for much of the previous week only once were categorized as transiently lonely; people who reported that twice were excluded from the analysis, highlighting the limitations of the transient‐chronic categorization for less clear‐cut cases. Using a slightly different approach, research among adolescents sometimes distinguished different loneliness trajectories over time [12, 15] using latent class growth analysis, for example [12]. For instance, Japanese adolescents who reported frequent loneliness at ages 10, 12, and 14, were categorized as chronically lonely; notably, 86.5% of them also reported frequent loneliness at the age of 16 years. Such different operationalizations highlight the current lack of consensus on what exactly chronic loneliness is defined by.

### 1.2 Possible Pathways Into Chronic Loneliness

Loneliness seems to increase people's focus on social cues, including a stronger focus on negative and threatening social stimuli [16, 17, 18, 19]. People who feel lonely have been found to interpret social situations more negatively, expect to be rejected or hurt by others more, and remember negative social situations more than people who do not report loneliness. While a focus on social cues has been suggested to facilitate social reconnection [18], a focus on social *threats* has been suggested to perpetuate loneliness [16, 17]. Accordingly, Dutch adolescents categorized as chronically lonely reacted more negatively to social exclusion and showed less enthusiasm in response to being socially included than people with other loneliness trajectories (e.g., with high‐decreasing or moderate‐stable loneliness) [15]. They also attributed social exclusion more to internal and stable characteristics, and social inclusion more to external circumstances. Negative perceptions, memories, and attributions of social situations can lead to behavioral patterns that perpetuate more negative social interactions (e.g., hostile or aggressive behavior) [16]. As such, certain cognitive patterns may increase loneliness both by shaping more negative perceptions of relationships, and by contributing to more negative *actual* social interactions.

**FIGURE 1:**
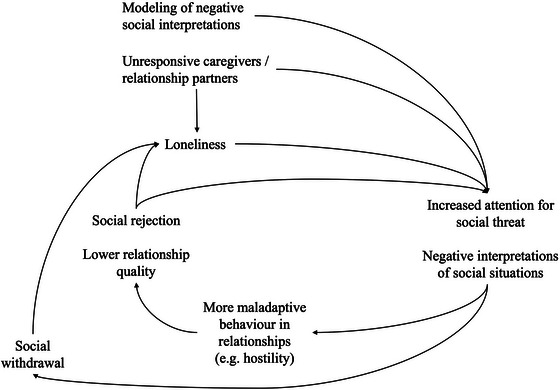
Possible intrapersonal processes underlying chronic loneliness.

Theorizing about such negative cognitive patterns may be important to understand how loneliness can be perpetuated, but it does not explain why only some people do not recover from loneliness or feel lonely all their lives. This may be explained by genetic disposition [20] or past relationship experiences, which can shape how people perceive, and act in, relationships [21, 22]. Furthermore, contextual characteristics may perpetuate loneliness by preventing that individuals can escape certain loneliness risks (e.g., unfulfilling relationships, stigma). Notably, while some risk factors—such as social rejection—are likely to be encountered by people with both transient and chronic loneliness, they may cause chronic loneliness if experienced earlier in life, repeatedly, or across various different relationships. The remainder of this section will describe some possible pathways into chronic loneliness (see Figure 1), providing a basis for the current analysis of perceived causes for chronic loneliness.

### 1.3 Past Relationship Experiences

#### 1.3.1 Insecure Attachment and Social Learning

Experiences with close others—especially in childhood—shape individuals’ expectations about social relationships [21, 23]. More specifically, if so‐called attachment figures are often unavailable or unresponsive, individuals learn that they cannot rely on others or that they will easily be rejected by them. Attachment figures are people an individual turns to when in distress and they include caregivers, romantic partners, or sometimes close friends. Unavailable or unresponsive behavior by attachment figures can result in preoccupation with others’ potential unavailability in times of need (*anxious attachment*) or rejection of closeness and attempts to rely solely on oneself (*avoidant attachment*). People with such so‐called *attachment insecurities* seem to have more maladaptive social behavior or negative perceptions of relationships [21, 24, 25], like those described for people who feel (chronically) lonely [15, 16].

For instance, they have been found to process social information in more pessimistic ways than people with more secure attachment [24, 25]. When asked to interpret relationship events, people with attachment insecurities were more likely to believe that their partner was rejecting closeness on purpose, and to attribute negative partner behavior as stable, internally caused, and global [24]. People with stronger anxious attachment feared relationship termination when facing relationship transgressions by their partner more [25]. People with stronger avoidant attachment more readily accessed instances of negative partner behavior [26] and had more pessimistic interpretations of their partners’ positive behaviors [25].

People with attachment insecurities also appear to manage conflict less adaptively than people with secure attachment [21]. People with more anxious attachment, for example, appear to more easily react with anger and despair if little support and closeness are provided [27, 28], to act in more intrusive and demanding ways, and to be more exploitable and submissive than people with less anxious attachment [29]. People with more avoidant attachment, by contrast, tend to be more competitive, less empathetic, and more socially withdrawn than people with less avoidant attachment. Accordingly, they seem to face more interpersonal problems, conflict, and lower relationship satisfaction than people with more secure attachment [21, 27, 30]. They report less stability in romantic relationships, and lower trust, less closeness, and less mutuality in friendships (for a review, see [21]). That is, people with more (vs. less) attachment insecurities seem to perceive relationships in more negative ways and to develop fewer fulfilling relationships, increasing the risk for chronic loneliness.

Notably, people may not only learn social behavior and interpretations from *reacting* to others’ behavior, but also by imitation [31]. For instance, if parents repeatedly attribute bad intent to others’ actions, children learn that, when in doubt, others’ actions signal hostile intent. This might trigger similar patterns of interpretation as attachment insecurities.

#### 1.3.2 Social Rejection and Not Fitting In

Past social rejection—for example, by peers—may also increase the risk for chronic loneliness by increasing people's sensitivity to rejection: they more easily perceive to be rejected in various social situations [22, 32, 33]. When perceiving to be rejected, people with rejection sensitivity seem to react with more anxiety, hurt, anger, hostile or aggressive behavior, social withdrawal, and jealousy or controlling behavior in partnerships [22]. Like insecure attachment or loneliness, rejection sensitivity may lead to perceptual and behavioral patterns that hinder the development of fulfilling relationships.

While occasional social rejection may contribute to transient loneliness, some people experience systematic or structural rejection because they are part of a stigmatized group or do not conform to prevailing social norms (e.g., sexual minorities) [34]. Together with feelings of being misunderstood or alienated from others, such repeated experiences of rejection may then result in chronic loneliness [35].

### 1.4 Contextual Characteristics

Contextual or societal characteristics may sustain loneliness if they prevent that people can reduce or avoid what causes their loneliness. For instance, cultural norms that discriminate against certain groups prevent that members of these groups can escape social rejection or devaluation, possibly hampering that fulfilling relationships can be established [34]. Furthermore, certain cultures offer few opportunities to create new relationships (low *relational mobility* [35]) or imply strong expectations to hold on to existing relationships (high *relational stability* [36]). This can prevent people from escaping unfulfilling relationships, which can perpetuate loneliness (e.g., disharmonious family relationships). People can also be prevented from escaping loneliness risks by immobility (e.g., through illness, age), particularly in contexts that do not provide suitable infrastructure and services for less mobile groups. More at the macrolevel, political or economic circumstances can force people to move away from close others (e.g., into exile; toward better‐paid jobs) or to stay in unfulfilling relationships (e.g., if living separately from family would be unaffordable). Additionally, such circumstances can cause continuous worry and mental ill‐being, and that may perpetuate loneliness [8].

## 2 Method

To (1) identify and describe different patterns of loneliness in people's lived experiences and (2) explore self‐perceived causes for chronic loneliness, existing qualitative data from two research projects were analyzed. The first project took place in 2019 and early 2020 [8]. Forty‐two participants ages 24–45 years from Austria, Bulgaria, Israel, India, and Egypt participated in in‐depth interviews to examine definitions, causes, and coping strategies for loneliness across cultures. Second, for a multicountry research project on loneliness and experiences of “not fitting in” with others (i.e., one perceived cause of loneliness [8]), we conducted in‐depth interviews with young adults ages 18–30 years [9]. In the current research, only 11 interviews from Turkey (collected in 2023) are included because data collection for other contexts was not yet completed or did not contain questions about loneliness. The first project was approved by the ethics board of the University of Groningen, the second project by the ethics board at Utrecht University.

See Table 1 for an overview of participant characteristics. The samples in this study were drawn from a broad range of cultural contexts, enabling an exploration of the causes of loneliness beyond a single cultural context. The original analysis of the data revealed few qualitative differences in how loneliness was defined or what was perceived to cause it across different cultural samples [8]. Additionally, data from the second project [9] aligned with those previous qualitative findings. This indicates that the mechanisms underlying loneliness may be qualitatively consistent across cultures, supporting the decision to pool samples in the current analysis.

**TABLE 1:** Sample characteristics.

	*n*	Age range	Gender			Relationship/family status		Education level		
**Project 1** (Heu et al., 2021)			Women	Men	Other	Single	In partnership	Minimum compulsory schooling	Secondary education/vocational training	University education
Indian sample	9	24–45	4	5	—	1	8	1	3	5
Egyptian sample	10	25–44	5	5	—	4	6	—	3	7
Bulgarian sample	8	27–44	5	3	—	1	7	—	—	8
Israeli sample	8	26–39	4	4	—	4	4	—	—	8
Austrian sample	7	26–45	2	5	—	2	5	2	2	3
										
**Project 2** (Heu et al., 2024)										
Turkish sample	11	19–28	8	3	—	5	6	—	2	9

In both projects, purposive convenience sampling was used, aiming for variation in gender, age, education level, relationship status (both projects), and minority status (second project [9]). To be eligible for participation, participants had to have grown up and currently live in the country where the data were collected, be a native speaker of one of the official local languages, and be between 25 and 45 years old for the first project [8] (although we also included one participant who was 24 years) or between 18 and 30 years for the second project [9]. They did not need to have felt lonely before. Participants were recruited through collaborators’ social networks (e.g., through social media posts, direct messages, and snowballing). Some prospective participants were also recruited through referrals by other participants or collaborators at the interview locations.

To obtain informed consent and participant demographics, participants filled in a brief paper‐and‐pencil questionnaire [8] or online survey [9]. Interviews in the first project were conducted offline, mainly by collaborators and principal investigator (PI) working together [8]. Interviews in the second project were conducted online and by a collaborator alone [9]. Interviews in Austria were conducted by the PI only. No differences in participants’ openness could be observed between online and offline environments. Interviews were transcribed and translated by collaborators, research assistants (RAs), or the PI. Importantly, participants in both projects were asked to report most recent and/or particularly intense experiences of loneliness, implying that participants often had more loneliness experiences than they reported.

**FIGURE 2:**
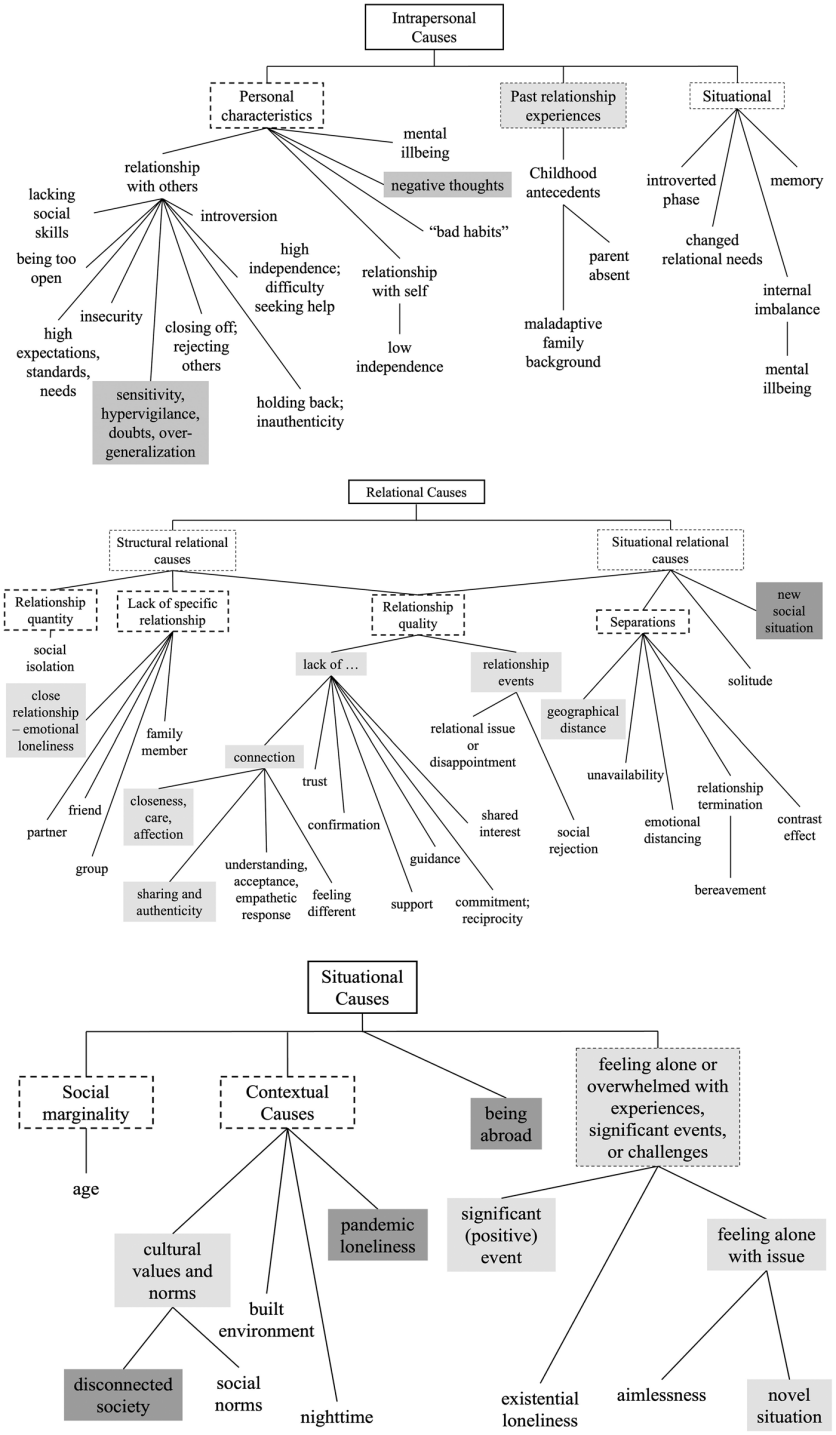
Coding trees with intrapersonal, relational, and contextual causes for loneliness (based on Heu, Hansen, et al., 2021) [8].

Data were analyzed in NVivo by the PI, a female assistant professor from Austria, living in the Netherlands. A qualitative content analysis was conducted, with deductive coding of loneliness causes based on Heu et al. [8] and inductive coding for new codes. For an updated overview of codes, see Figure 2. Based on broad conceptualizations of chronic loneliness in the literature (see Introduction section), the PI classified each interview as an example of transient versus chronic loneliness. However, rather than just two, multiple loneliness trajectories emerged—transient, recurrent, prolonged, and chronic (see Results section). Based on a preliminary description of these patterns, both the PI and a RA—a male British master student in psychology—classified each interview again. The RA was less familiar with the loneliness literature and had not been involved in interviewing, adding a different perspective on the data.

Only in three out of 53 cases did one rater classify a case as chronic loneliness while the other classified it as transient loneliness. However, there were multiple disagreements on “adjacent” patterns (e.g., chronic loneliness vs. recurrent loneliness; see Results section). Differences were resolved through discussions, which helped define different loneliness patterns more precisely. Using these final definitions, the PI classified interviews once more herself. Table 2 offers an overview of the distribution of participants across categories; the reasoning for the categorization of each case and illustrating quotes are provided in Table S1.

**TABLE 2:** Number of participants and occurrence of selected codes in each category.

	Transient loneliness	Prolonged loneliness	Recurrent loneliness	Chronic loneliness
Number of participants	20	6	16	11
**Perceived causes for loneliness**				
Distant, troubled, or lacking family relationships	0	1	0	6
Lacking closeness and affection	3	1	3	5
Feeling different/not fitting in	11	2^1^	7	6
Sensitivity, hypervigilance, rumination, and overgeneralization in relationships	3	0	5	3
Relational issue or disappointment	2	1	7	3^2^
Expectations and ideals	3	0	3	6
Discomfort with oneself	0	0	5	5

*Note*: Numbers only serve as a rough indication of the distribution of reported causes and need to be interpreted with caution due to the small sample size. Notably, some participants experienced different loneliness patterns in different phases of their lives. Their interviews were categorized, and causes were analyzed for their most long‐lasting or recurrent pattern, which the interviews also tended to focus on. For instance, a participant who had experienced chronic loneliness earlier in life and recurrent loneliness later was predominantly interviewed about chronic loneliness and analyzed as an example of chronic loneliness. ^1^Two or ^2^one additional participant(s) in this category also mentioned this loneliness cause, but as a cause for transient loneliness in a different period of life.

## 3 Results and Discussion

### 3.1 Loneliness Types

Rather than only two, the interviews revealed several distinct types of loneliness experiences: transient, prolonged, recurrent, and chronic. Each type will be discussed in more detail below.

#### 3.1.1 Transient Loneliness

People with *transient* loneliness typically experienced loneliness in response to specific events or experiences (e.g., conflicts, break‐ups, problems, bereavement), lasting a few minutes up to one or—rarely—2 years (in line with [11]). Notably, such transient loneliness experiences differed greatly in intensity. Whereas most people reported relatively low intensity, some people reported strong suffering.

I used to even enjoy the feeling of being alone in the past. But now, it's a feeling that overwhelms me. […] Sometimes, I feel very lonely even within a relationship, especially in my romantic relationship. […] Just last week, for instance, I experienced an overwhelming sense of loneliness. I didn't know what to do; I felt trapped. (T2, female, 26, Turkish)

And I can consider the 4‐month period when I went to the Netherlands for my master's in 2018, and the 3–4 months after I returned, as a period when I felt lonely. It was about 7–8 months in total. […] So, when I look at that period, it feels like a kind of self‐erasure in my life, like a black hole, the deepest point of loneliness. (T1, male, 28, Turkish)

These quotes suggest that—despite recovering relatively quickly—people with intense transient loneliness experiences may sometimes benefit from external support.

#### 3.1.2 Prolonged Loneliness

Many participants reported feeling lonely for multiple months after life‐changing events (e.g., bereavement, losing a close other) or due to difficult external situations. However, some participants reported unusually long periods of loneliness (i.e., more than 2 years). For instance, one participant described having been feeling lonely for 5 years, since her father's death. Albeit situational, such “prolonged” loneliness experiences were thus longer than what most people with transient loneliness reported.

I was very in love with one of my classmates—for many years, for 7 years. I was so in love with him that I was feeling extremely alone even though I was surrounded by people, friends, but I wanted only him and nothing else in the world could replace his love. (B1, female, 44, Bulgarian)

Notably, people with such prolonged experiences typically reported transient (sometimes recurrent) loneliness throughout the rest of their lives.

#### 3.1.3 Recurrent Loneliness

A relatively large group of participants reported experiencing loneliness once every couple of weeks or months—more frequently than people with transient loneliness, but less frequently than people categorized as chronically lonely. Indeed, people with chronic loneliness seemed to feel lonely every couple of hours or days (if not continuously).

I would say that it is something that is systematic. It inevitably happens all the time, in specific situations. Even if I look back, there have been moments in my life when I have had more frequent periods of loneliness, but again, it was not 24/7 loneliness. Or, at least, it was not prolonged—even if it's 24/7, it's due to something, an event. Something that made me feel lonely. Very rarely this will continue permanently, for a long time. (B2, male, 33, Bulgarian)

Notably, reports by participants with recurrent loneliness suggested a personal tendency to react with loneliness to various situations (see also Table S2). This is different from most transient loneliness experiences, which seemed more bound to concrete external situations.

Maybe, in most cases, the reason is really that it's due to communication with other people, due to life with others. […] I mean, one way or another, when you feel rejected, rejected from society, rejected from other people, that is when you feel lonely. You can feel misunderstood or excluded from that particular society in many different ways and that is when loneliness occurs. (B2, male, 33, Bulgarian)

There are simply points in life where one maybe does not feel understood and where this kind of makes one withdraw. And where one maybe unwillingly creates a certain loneliness through that. […] It certainly also depends on one's psychological stability. If one rather does not feel very stable anyway and is drifting into a depressed episode, these phases of loneliness are clearly longer than if one is actually quite stable anyway, and one just gets kicked out for a bit. Then it's maybe just for an hour or so. (A8, female, 35, Austrian, recurrent loneliness)

#### 3.1.4 Chronic Loneliness

Participants who were categorized as chronically lonely reported having been accompanied by loneliness for their entire lives or large shares of their lives, usually with an onset in childhood or adolescence.

Um, loneliness is something I struggle with a lot. I wouldn't—I can't remember a single point of time where I did feel lonely. I just can't remember not being lonely. (IN2, female, 24, Indian)

Well, in general, I always feel lonely. […] maybe the intensity within 3 days puts a curtain in front of this feeling, but at the end of the day, it's as if nothing can take away that loneliness. I always feel like I'm left to myself. (T11, female, 25, Turkish)

Multiple participants with chronic loneliness described that their loneliness was not continuously present, but more perceptible in specific situations.

It has actually been stronger and weaker in waves, but has latently been there. […] Sometimes, it's more strongly perceivable, sometimes it's less strongly perceivable. (A6, male, 33, Austrian)

This is in line with previous studies that operationalized chronic loneliness as *some level of loneliness* at multiple consecutive time points [6, 7, 13, 14].

### 3.2 Perceived Causes and Mechanisms for Recurrent or Chronic Loneliness

**FIGURE 3:**
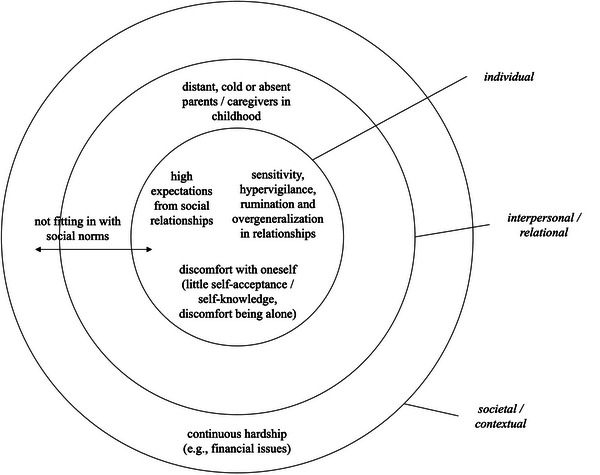
Perceived causes for chronic loneliness in current research.

People reported feeling lonely because of specific events (e.g., separations, lacking a partnership, facing problems, bereavement) across all loneliness patterns. However, while transient and prolonged loneliness were typically linked to specific, external events, recurrent and chronic loneliness were more often attributed to early relationship experiences and (acquired) intrapersonal tendencies. Specifically, both chronic and recurrent loneliness were attributed to negative perceptual or thought patterns in relationships, or to discomfort with oneself. *Chronic* loneliness was often attributed to unfulfilling relationships with parents in childhood, high expectations from social relationships, or systematically not fitting in with social norms (see Figure 3). These possible causes of recurrent or chronic loneliness will be discussed in more detail in the following. Although numbers in this qualitative research need to be interpreted with caution because of the small samples size, Table 2 provides an overview of how often these themes occurred for each loneliness pattern.

#### 3.2.1 Unfulfilling Relationships With Parents in Childhood

In line with the notion that chronic loneliness can result from attachment experiences in childhood [21], more than half of participants with chronic loneliness explained their loneliness by maladaptive relationships with their parents when growing up. No participant with transient loneliness did. Experiences included little emotional connection to parents, absence of a parent, strict or emotionally cold parenting, or emotional or physical violence.

Well, I could, through therapy and so on, […] recognize the feeling from my childhood. So, the feeling is similar or equal, that was just not clear to me. […] Yeah, psychological and physical violence, neglect, uh accusations, insults, humiliations […] The loneliness and withdrawal start there, right? (A6, male, 33, Austrian, chronic loneliness)

And I also learn much about what I haven't got during my childhood or—because I believe that this is always a key to loneliness. […] My mother was very stressed and had many different fears and existential fears, which then made me feel rejected—although she loved me for sure, but I didn't get it in the way that she might have been able to give it if she had been in a better life situation. […] At home, there was only stress—and much fear. And much darkness, and—not nice. I didn't want to go home, I didn't want to go to school—so, I didn't have a connection to anything. […] Only later, I understood that—that I was actually often longing for a father … and that this was the reason for much of my loneliness. (A1, male, 35, Austrian, chronic loneliness—recovered)

This could be felt as a lack of closeness or safety even in adulthood, often perceived as direct cause for loneliness rather than necessarily through maladaptive perceptions or behavior in current relationships (see Table S2 for additional quote).

I've had a very uh tumultuous relationship with my family, and it always makes me crumble. […] And um, they've been incredibly strict. It's a very authoritarian um household. And I wasn't given a lot of affection, physical affection and all of that. So, I find myself on most occasions feeling touch stuff—I uh [laughs] want to hold hands with my friends or lean my head on their shoulder. Small little um … feelings that let me know that I'm not feeling—I'm not that alone and there's someone who cares about me. (IN2, female, 24, Indian, chronic loneliness)

And my mom is a very serious person. […] So, I think, this constant feeling that the atmosphere at home was not very maternal or supportive, and that I had to be incredibly independent. […] I think that many things resulted from these feelings that I need to manage my life on my own, because there is no one else. […] I always felt distant from my parents and I was fearful of what they would say and what they wouldn't say. […] And this made me feel really lonely. (I4, female, 26, Israeli, chronic loneliness)

Relatedly, although almost all participants attributed their loneliness to some shortcoming or issue in their relationships, those with chronic loneliness seemed to miss closeness, care, or affection more intensely or in more different relationships—rather than in only one specific relationship—compared to those with other loneliness patterns.

#### 3.2.2 Not Fitting In

Across loneliness patterns, people reported not fitting in with others around them as a cause of loneliness (see Table S2 for quotes). However, while people with transient loneliness reported specific situations (e.g., not fitting in at a party; see Table S2), people with chronic loneliness reported *structurally* not fitting in (e.g., with societal norms).

I just think that I felt really alone in the sense that I felt like I am the different one every time that people saw me as that different one and treated me like that. […] And all my life, I've been feeling it. […] We have a WhatsApp group of the whole class, and then there is a group for the guys and the girls. And I'm in neither of them. […] So, like, from the perspective of the different genders maybe, in my case, that's what brought the sense of loneliness. (I3, male, 27, Israeli, chronic loneliness)

Yes, you see, and there my ADHD comes in now, ja? Um, I've always had the feeling that I was a little different, ja? And that was already lonely because people didn't understand what I explained, ja? What I wanted to tell them. They didn't understand, ja? that I think differently, that I am different, ja? That I feel differently. […] And I actually wanted to get among people more. You know—but somehow they did not accept me really. Or I was too extreme. (A5, male, 41, Austrian, chronic loneliness)

#### 3.2.3 Sensitivity, Hypervigilance, Rumination, and Overgeneralizations in Relationships

Reports of unfulfilling relationships among people with chronic or recurrent loneliness (see above for relationships with parents) may be interpreted as information about relationship quality, but they may also indicate possible perceptual tendencies [16, 18, 21, 22]. Indeed, about a third of the participants with chronic loneliness and about a third of participants with recurrent loneliness reported high social sensitivity—a higher proneness to perceiving social cues, and stronger reactions to them—or excessive thoughts about own and others’ social behavior when asked about the causes for their loneliness:

I overthink to death, I always overthink. I overthink every conversation I've had—I'm overthinking right now. And it's something that I can't stop. And I think it has a lot to do with how aware I am of myself, of what I'm doing when I'm around people or even by myself. (IN2, female, 24, Indian, chronic loneliness)

I mean, the main reason for this [feeling lonely] is generally that I scrutinize people's behaviors too much and they seem artificial to me. Including my own. My behaviors toward others seem artificial. It's like playing a role, acting. (T3, male, 26, Turkish, chronic loneliness)

Similarly, one participant with highly intense current loneliness (long‐term pattern still unclear) reported a strong tendency to overthink social relationships (see Table S2 for quotes). By contrast, the only two participants with clear transient loneliness who reported negative perceptions of social relationships did not seem to suffer much from, and rarely experience, these perceptual tendencies (see Table S2 for quotes).

In line with notions of rejection sensitivity [22] or hypervigilance to social threat, the negative thought patterns that participants described often coincided with a fear of being rejected, doubting others’ trustworthiness, or overgeneralizing negative relationship experiences.

Even though there are people with whom I can share, but I'm scared because sometimes they might misuse information that you provide them, or they might take advantage of what you share. So, I'm always scared. (IN1, female, Indian, recurrent loneliness)

When I can't explain my problems to someone, I conclude that nobody understands me, nobody loves me, and that makes me very lonely. (T6, female, 26, Turkish, recurrent loneliness)

There is such—such a basic feeling of mine, that relates to my childhood, gell? That is the latent feeling to be—in social groups—well in groups, to be accepted at most. [I feel like] to belong to a group, I have to work more than anyone else and each minimal mistake is immediately uh the […] “exclusion from the group” […] That does not happen consciously […] When I go through it cognitively, yeah, it's not possible […] So, this feeling to be tolerated at best. (A6, male, 33, Austrian, chronic loneliness)

For example, I don't get close to anyone since childhood. I mean, I don't develop a close bond with anyone even if I study and work somewhere for many years. I keep my distance from everyone; I'm a reserved person who sets boundaries. […] this is the result of what happened [repeatedly feeling different from others]. I got used to isolating myself a bit and kept myself separate. (T9, female, 27, Turkish, recurrent loneliness)

The last two quotes above highlight that some participants linked their negative thought patterns to family relationships in childhood, in line with the idea that negative relationship experiences with caregivers in childhood lead to maladaptive patterns of processing relationships [21].

People with recurrent loneliness, in particular, described recurrent relational issues or disappointments as triggers for loneliness (see Table 2). However, based on their narratives, they seemed to be often relatively sensitive to disharmonies or face others who sought conflict, rather than necessarily to engage in more conflict than people with other loneliness types (cf. [16, 19, 21, 22]; see Table S2 for a possible counterexample).

For sure, very often that negative feeling of loneliness could come from interaction with the people most dear to you. Friends, family, and so on. […] Because these people are that dear to you, their rejection comes very hard on you. If they don't accept you somehow, this will have the strongest emotional influence on you. […] And of course, you could feel very lonely due to the dynamics and the communication between you and your partner. (B2, male, 32, Bulgarian, recurrent loneliness)

I mean, there will be a lot of problems at home—I won't be able to tell them to others. Even if I told others, I don't believe that they would be of help to me. That's why I feel lonely. […] Like, I go to work ‐ at home, they [relatives] ask for money. […] I mean, when they come and ask [how I am doing] once a month, I'm fine. But again, when they ask for money, it is a problem. Then, I feel lonely. (IN6, male, 34, Indian, recurrent loneliness)

#### 3.2.4 Expectations and Ideals

More than half of the participants with chronic loneliness reported perceiving highly demanding ideals and expectations from their relationships as loneliness cause, as compared to only three out of 20 people with transient loneliness (one of them with intense current loneliness, see Table S2 for quotes by the other two participants). Participants with chronic loneliness or recurrent loneliness reported having ideals that were more difficult to obtain (e.g., finding a soulmate) and holding on to these ideals relatively long (e.g., wishing for a soulmate for years rather than only in a moment of being misunderstood).

I would always think: […] I would so much like to have someone […] with whom I just really connect and who is a 100%—like, best friend, or something like that—so a 100% always by my side […] and because I always wanted to have that, but somehow never got it […] I always kind of felt lonely […] somehow also when I was together with other people because—through different inner convictions and thoughts […] “Yeah, I could now have—no clue… She or he is not really in tune with me.” and somehow: “Is it really of advantage for me now to be […] with him or uh her? […] What's the point of that?” […] but that's just nonsense somehow. (A9, female, 26, Austrian, recurrent loneliness)

I'm not saying uh… my—my boyfriend tried—ex!—he tried to be caring, but it wasn't the same way I wanted it. So, I tried putting this ideal image that I had in my head into everything that he did and it wasn't enough and—I realized I was battling with myself about what I wanted. (IN2, female, 24, Indian, chronic loneliness)

Research in sociology and media studies describes an idealization of romantic relationships in Hollywood movies, possibly creating unrealistic romantic ideals [37]. This may be particularly relevant in secular societies where romantic relationships are expected to address the need for meaning, transcendence, and belonging that were earlier catered to by religion or religious communities [38, 39]. Similarly, idealized portrayals of friendship in drama series may create a longing for unrealistically close friendships [40]. To understand the development of chronic loneliness, the key question is who is prone to adopting and holding on to such ideals. For instance, an absence of belonging, safety, and affection early in life, such as in family relationships, may result in higher perfectionism [41] (implying more demanding expectations) or possibly make individuals seek safety in the idea of a perfect relationship.

[…] when there was a problem in the family—a conflict between parents—and so, the child somehow perceives it. At least I perceived it this way—that this is an example that I shouldn't follow. That is, I have to look for the person for me and to build a healthy relationship, so that this thing doesn't happen. And this sometimes made me act very desperately because I look and look and look for such a person but I don't find them […]. (B6, female, 27, Bulgarian, chronic loneliness—recovered)

[My loneliness could be due to] things I have missed since I was young, things I could not get. […] You know, there are people that are always searching for someone that complements them—their other half. So, I feel this way. I feel that there is a lost part of me that I'm always searching for. […] I think that he is mainly the partner, but he will also play all these roles. He will play the role of the friend, of the father… (E7, female, 35, Egyptian, chronic loneliness)

Strong relationship ideals or expectations may also flow from high trait perfectionism, which seems to have both a genetic and environmental basis [42]. Future research with twin study or longitudinal designs, assessing family relationships in childhood and relationship expectations in adulthood, may help better understand who is prone to developing long‐lasting relationship dissatisfaction through overly high expectations.

#### 3.2.5 Discomfort With Oneself

Multiple participants with recurrent or chronic loneliness (but no participants with other loneliness patterns) traced their loneliness back to lacking knowledge about their needs and preferences, insecurity communicating these, little self‐acceptance, or discomfort being alone (see Tables 2 and S2).

Like, you think that you are a certain person, someone from the outside comes along, they change you a bit—especially if you're a bit more vulnerable, weaker. You start forming a different personality to be similar to them, to fit in. And at some point, you drift away from yourself and you're completely different. Like, you can't recognize yourself. And when a person drifts away so much from themselves, this loneliness is very hard to fix. […] But when you find it [true self], then a person doesn't fall into such moments of loneliness because they know that they can always have a talk with themselves in peace, if necessary. (B6, female, 27, Bulgarian, chronic loneliness—recovered)

I'm someone who struggles a lot with being on my own, and when I think too much, I continue by creating scenarios and going over things like “this happened, that happened”. And I'm also merciless to myself, honestly. That exhausts me a lot. (T11, female, 25, Turkish, chronic loneliness)

These self‐reports fit findings that lower self‐esteem can predict more loneliness [43, 44], possibly perpetuating loneliness. Multiple participants who talked about a weak relationship to themselves also mentioned difficult relationships with their parents in childhood, which is in line with the idea that children with secure attachment relationships with their parents are more likely to develop higher self‐esteem [45].

Notably, most participants who reported discomfort with themselves were young women, aligning with findings that traditional gender roles assign less assertiveness or agency to women than men [46], and findings of lower self‐esteem in women than men [47]. Furthermore, all participants who reported those causes for loneliness had also recovered (at least partially) from their chronic or recurrent loneliness. This may suggest that people are often unaware that discomfort with themselves could be a cause of their loneliness—possibly because discourse tends to attribute loneliness to a lack of social connection.

#### 3.2.6 Contextual Restrictions to Escaping Unfulfilling (Social) Situations

Although cultural norms were sometimes mentioned as causes of loneliness (e.g., competitiveness) or as influencing how people dealt with loneliness (e.g., norms not to share family issues outside the family), they did not seem to play a clear role in people's explanations of chronic loneliness. Generally, participants did not report contextual influences on their loneliness much. However, one Egyptian participant reported continuously feeling lonely because of financial difficulties, in line with findings of more loneliness in groups with lower socioeconomic status [48, 49]:

Well, if you find something that makes you happy in life or something that pulls you out of hardships or problems, that's when you don't feel lonely. But if there are problems or difficulties in your life, you'll feel lonely. (E6, male, 40, Egyptian, chronic loneliness)

### 3.3 Perceived Reasons for Recovery

Because some participants reported having recovered from chronic, recurrent, or prolonged loneliness, reasons for recovery were explored to better understand how such experiences of loneliness could be reduced. For one, in line with findings on discomfort with oneself as a perceived cause of loneliness (see above), several participants—mostly women—reported a reduction in their loneliness after gaining a better understanding of their needs, communicating those needs more effectively, accepting themselves, or recognizing that others did not need to fulfill all their ideals (see also Table S2).

It sort of took me time to figure out that I was repressing, you know, some of my wants and needs, so—sometimes it would come out—like, I—I would sort of uh—I would sort of have these outbursts of emotions that I wasn't even exactly expecting, because I held everything in too long and then um—I think that the more that I learned to express uh my feelings and—and my wants and needs in real time, so—uh—so that feeling [loneliness] just basically goes away. (I1, female, 39, Israeli, recurrent loneliness—recovered)

I definitely don't feel that I can connect better. […] But I'm more—I've become more accepting toward myself. (IN10, male, 28, Indian, recurrent loneliness—recovered)

Relatedly, one participant described a decrease in her loneliness after she opened up about her sexual orientation (see Table S2).

While one participant perceived that his loneliness had decreased as he became more extraverted, several others reported a reduction in their loneliness only after they became more comfortable with spending time alone (see also Table S2) and when they started avoiding others who they did not feel connected to. Their initial attempts to reduce loneliness by surrounding themselves more with people had been unsuccessful.

If I'm somewhere with friends, I prefer, if I don't feel okay, to leave, to stay by myself for a bit, because this [being with friends] just prolongs my agony, so to say. That is, I feel lonely, but if someone is constantly saying things around me but inside I have some thoughts that I want to deal with—and at some point, this chaos of internal and external voices begins and… I prefer to remain alone. […]

There was a point when I was very discouraged about what my environment was and I constantly surrounded myself with new people, I tried one environment, I tried another, I was straight up, “scattering” myself. […] Until at some point, I started practicing “hygiene” toward the people close to me. […] Like, now, years later, I have fewer friends, I see fewer friends, […] but, um… I know that we are very close and I feel very comfortable with them. I've never felt lonely with them. […] (B6, female, 26, Bulgarian, chronic loneliness—recovered)

Several participants—particularly men—also reported recovering from loneliness through external changes (see Table S2 for quotes). Participants with prolonged, recurrent, or chronic loneliness in young adulthood sometimes reported feeling less lonely after getting married. One participant reported that his chronic loneliness had fully vanished only when he became a parent in his 30s. Another participant reported that the loneliness he had always felt in his family environment reduced significantly when he moved to a boarding school.

## 4 Implications

By describing different loneliness patterns in people's lived experiences, this study provides the basis for future research on the types of loneliness that warrant treatment. The findings suggest that future research should examine not only long‐lasting loneliness, but also loneliness that keeps recurring in different situations, or transient loneliness that is overwhelming in intensity. By identifying possible risk factors or underlying mechanisms—particularly for chronic and recurrent loneliness—the current findings may also serve as a basis for future research aimed at developing targeted interventions. Indeed, interventions that specifically target causes of loneliness *that require treatment* are likely to be both more effective and cost‐effective than interventions targeting any loneliness experience.

For instance, interventions currently often aim to increase social contact, which is neither a common cause for chronic, recurrent, or prolonged loneliness in the current study among young and middle‐aged adults; nor did it clearly distinguish chronic or recurrent from transient loneliness. Relatedly, findings seem to contradict the idea that chronic loneliness can generally be prevented by counteracting causes of transient loneliness, assuming that chronic loneliness is essentially a transient loneliness experience that people cannot recover from. Unlike those with transient loneliness, participants with chronic loneliness typically described the onset of chronic loneliness in childhood, often as early as they could remember.

Indeed, most participants with recurrent or chronic loneliness seemed to feel lonely because of a personal or acquired vulnerability (e.g., through relationship experiences in childhood). In contrast, participants with transient or prolonged loneliness seemed to feel lonely due to single external events or situations. If confirmed in future research, chronic and recurrent loneliness may be addressed by reducing that personal vulnerability. For instance, psychotherapy [50] may help process unsupportive childhood relationship experiences, change maladaptive thought patterns about relationships, or increase self‐acceptance, and communication of one's needs to others. Parenting programs might prevent negative attachment experiences. In addition to support groups for marginalized groups, interventions focused on destigmatization could help prevent repeated negative situations of not fitting in.

## 5 Limitations and Future Directions

While qualitative research captures the lived experiences of loneliness, individuals may not always be able to accurately identify the true causes of those experiences. For instance, they are typically unable to report subconscious processes (e.g., perceptual biases) and may consider personal tendencies or experiences “normal” that would be judged as maladaptive or extreme from the outside. For instance, people may not know that they have high expectations of social relationships. Vignettes [24, 25] or experiments may help more systematically study thought patterns underlying long‐lasting, recurrent, or intense loneliness.

Relatedly, the finding that people with chronic loneliness can often trace their loneliness back to childhood relationship experiences does not necessarily mean that those were the actual causes of their chronic loneliness. For instance, interactions may be perceived more negatively by people with higher genetic social sensitivity [51], which may also perpetuate loneliness. Furthermore, learnt narratives about loneliness influence which loneliness causes people identify. People who experience recurring negative feelings are more likely to seek psychological help than people who are doing well, and revisiting relationships with parents—or other relevant caregivers—is a central component of many approaches to psychotherapy (e.g., psychodynamic, systemic, or schema therapy). Although not explicitly asked, five out of 11 participants with chronic loneliness mentioned having seen a psychotherapist or psychologist to address mental health issues, such as depression. This contrasts with only three out of 20 participants with transient loneliness and two out of 16 participants with recurrent loneliness. This may not be surprising given the strong correlation between loneliness and mental health [2]. While participants did not seek help specifically for dealing with loneliness, they may have addressed these feelings during therapy or counseling. Because theorizing and empirical research suggest a strong influence of family relationships on various psychological outcomes [22, 52, 53], participants’ perceptions that their family relationship experiences contributed to their recurrent or chronic loneliness probably reflect reality to some extent. Nevertheless, studies with a multi‐informant approach (e.g., interviewing different family members) or observation techniques would be relevant to assess to what extent participants’ perceptions reflect actual family dynamics. Additionally, longitudinal studies following individuals from childhood (when they are arguably influenced by family relationships) to adulthood (when they arguably feel the consequences as chronic loneliness) would need to test that link.

Finally, in both studies in this reanalysis, participants were asked to provide *examples* of loneliness experiences. Consequently, the causes of loneliness they reported were not necessarily exhaustive. For instance, participants who did not mention maladaptive family relationships had not necessarily experienced satisfying ones. They may simply not have reported family relationships. Qualitative in‐depth interviews that chronologically walk through people's lives may help understand the sequence of, and interrelations between, different loneliness risks. Furthermore, correlations between loneliness risks may be better understood through questionnaires with comprehensive lists of loneliness causes. Such questionnaires would also allow to compare prevalence and predictive strength of loneliness causes across loneliness patterns in representative samples. In the long run, this may help develop interventions targeting most prevalent and most predictive loneliness causes in specific groups that require support to deal with their loneliness.

## Author Contributions

The author confirms responsibility for study conception and design, analysis and interpretation of results, and manuscript preparation.

## Conflicts of Interest

The author declares no conflicts of interest.

## Supporting information




**Supplementary Table**: nyas70082‐sup‐0001‐tableS1.docx

**Supplementary Table**: nyas70082‐sup‐0002‐tableS2.docx

## Data Availability

The data that were analyzed in this study can be requested from the corresponding author, but are not publicly available to protect the privacy of research participants.
