# Supplementary material for: A Moment Versus a Lifetime: Patterns of Loneliness and Perceived Causes in People's Lived Experiences
Source: Ann N Y Acad Sci. 2025 Oct 3;1553(1):172–86. doi: 10.1111/nyas.70082 (PMC12645267; doi:10.1111/nyas.70082)
Supplement: Supplementary file 1 — Supplementary Table: nyas70082‐sup‐0001‐tableS1.docx [file NYAS-1553-172-s002.docx]

**Table S1**

|  | **Loneliness Type** | **Motivation** | **Example Quote** |
| --- | --- | --- | --- |
| A1 (male, 35) | Chronic loneliness (recovered); currently transient loneliness | Describes having felt lonely from childhood until his 30s because of distant or absent parents | I think that I have really felt lonely during my childhood. And then, for a very long time. And I don’t think it was because my parents didn’t love me, but – I think that all – everyone loves their children. But many people are not aware that they are not able to properly communicate their love - or to pass it on or have not learnt to express it openly. And that they are actually lonely themselves, and, as such, hand down their loneliness. […] I think that I have really felt lonely during my childhood. And then, for a very long time. And I don’t think it was because my parents didn’t love me, but – I think that all – everyone loves their children. But many people are not aware that they are not able to properly communicate their love - or to pass it on or have not learnt to express it openly. And that they are actually lonely themselves, and, as such, hand down their loneliness. […] I would say until I – around between 16 - 16 and 20, I started to somehow create my identity anew and to have new friends and, therefore, a new family, a new circle ... how should I say – established new contacts, and then maybe, for the first time, I felt less lonely. But really not lonely anymore I’ve only been feeling in the past three or four years. And really really not lonely since 1,5 years.  I was - for instance, during primary school, I have - one is - I was among there. I felt very lonely there because I was a foreigner in - in - and one made me feel that. I don't believe that the children did this on purpose, but society does that, and the children hence do that, too. And I didn't have safety at home, no back-up. At home, there was only stress - and much fear. And much darkness, and - not nice. I didn't want to go home, I didn't want to go to school – so I didn't have a connection to anything. And - and - there were loads of people around me, but I felt misunderstood by all of them and, hence, lonely, because - I think that this is what loneliness is. If one feels misunderstood, or not accepted, or – wrong. |
| A2 (male, 45) | Transient loneliness | Reports hardly any loneliness experiences (only as small child) | Lonely ... Yeah, in the time when I was a small child. That is ... I assume, approximately between one and - or yeah - one and two. So, child's bed, bed time, I think, that was ... Otherwise, I can't remember, no. |
| A5 (male, 41) | Chronic loneliness (possibly partially recovered) | Describes having felt lonely since childhood because of feeling different (e.g., due to having ADHD, migration background); reports having withdrawn due to not feeling understood | For ten years, I haven't really had any friendships except for one - but that - that has always been quite sporadic. Every three or six months, we have maybe met up once or we chatted, ja? It ... I have been withdrawing since 2012 or so, I believe - well okay, it's been seven years, not ten […]  I've always had the feeling that I was a little different ja? And there it was already lonely because people didn't understand what I explained ja?  Exactly, this is what I am discovering right now. That I withdraw and don't feel lonely. Now, I am discovering exactly this. But earlier, it was different for me. Exactly, that withdrawal is loneliness. And I actually wanted to get among people more. You know - but somehow they did not accept me really. And I was too extreme, respectively.  Yes... yes, [I felt lonely] when I thought I had a relationship with someone and it didn't work out and she actually left me and didn't want to hear from me anymore, totally ignored me and yeah, there I was really lonely. Actually, I wanted to actually commit suicide that day. So that was quite intense. […] Oooh, that lasted long... […] Three months, something like that. Yeah, because I also broke off contact with my parents then and all kinds of things... […] |
| A6 (male, 33) | Chronic loneliness | Onset of loneliness in childhood with difficult family relationships; describes having felt lonely with fluctuating intensity ever since; describes social anxiety and social islation; difficulty finding partner | [I felt lonely for] practically the entire last year. […] But that is because, I am – I am to a certain extent a social phobic. I don’t know whether one can say I’ve always been that, ja? Uhh, depressions have hit me quite strongly in the past couple of years. […] Um yeah, and then that is kind of a - comorbidity I say now, that loneliness is kind of always – at least in some interaction, ja? With social fear and depression, which fertilize each other, and that also adds to that. So … it is difficult to identify a specific situation or a – a moment - it has actually been stronger and weaker in waves, but has latently been there.  Well, I could, through therapy and so on, and different possibilities that one has, pinpoint to recognize the feeling from my childhood. So, the feeling is similar or equal, that was just not clear to me. […] Therefore, it’s difficult to tell when it – it was maybe a little less, in a certain time period, but it has its origin somewhere […] at 8, I can pinpoint it relatively well. […] Yeah, psychological and physical violence, neglect, uh accusations, insults, humiliations – so, a pretty- pretty terrible programme. I was, however, never aware that that actually – how terrible that actually is, gell? It is like – it was always there, bearable for me, it’s just, that was natural. […] Sometimes, it’s more strongly perceivable, sometimes it’s less strongly perceivable. And then maybe also confusable with depression […] |
| A7 (male, 28) | Transient loneliness | Describes that he hardly ever feels lonely; reports only transient loneliness experiences without being entirely sure if he really felt lonely then (e.g., when no one could help him with health problems) | Um, no. Well, I - I myself have actually seldom the feeling that I - well, I cannot remember that I would ever have felt really lonely. So no, I don't really, no.  I've always had a relatively large friendship circle. And for some time, that was maybe a little more difficult back then, but I've never had the feeling that I was - was entirely alone in the world, yeah, and because I also have two sisters and - and, um, also a relatively large family, there was never that feeling that there is no one there.  Or, now I remember, if I think about it - with - with friends or acquaintances who still like to drink at my age and if one, for example, doesn't join for drinking that much, then it can, for example, happen that, on that evening [halt], one has a slight loneliness, simply because - because you don't belong at that moment [ja?]. |
| A8 (female, 35) | Recurrent loneliness | Reports multiple different situations of loneliness in everyday life, despite close relationships, suggesting some susceptibility to feeling lonely | I am still studying, and I really have many people around me - but that is no guarantee that one is not lonely. There are simply points in life where one maybe does not feel understood and where this kind of makes one withdraw. And through that maybe unwillingly creates a certain loneliness.  Interviewer: The experience with the death of your father - that was probably longer then.  A8: Erm, more frequent. Since it is, after all - when one lives in a family and the children are partly - I mean, the youngest is now 11 [years old], back then he was still in primary school - you actually rush from one event to the next. Also with studying, working, school and so ... One also distracts oneself and that shifts the loneliness a little. But then there are silent hours - for instance, during the night - when everyone else is sleeping and you yourself just cannot sleep - where one feels a little lonely. Even though it is not necessary.  It certainly also depends on one’s psychological stability. If one rather does not feel very stable anyway, and is drifting into a depressed episode, then these phases of loneliness are clearly longer than if one is actually quite stable anyway, and one just gets kicked out for a bit. |
| A9 (female, 26) | Recurrent loneliness | Reports having felt lonely many times in her life, also in close relationships; reports personal perceptions and convictions as basis for many loneliness experiences, suggesting some susceptibility to feeling lonely | So, I feel lonely time and time again. Although I am, for instance, in a relationship or kind of - but I feel lonely time and time again because one is not always that open and like "wow, now I want to talk to a hundred people" or the relationship is currently not at its absolute best and one distances oneself and, like - and then one also just feels lonelier.  When one has been hurt or if one kind of feels hurt or something like that. Or if it currently doesn't fit like one imagines it or something like that. Then one rather withdraws, for instance, and um, feels lonelier somehow than when one is kind of open and happy and so - yeah, so ... I believe that these are really, well inter-, well loneliness, for me, has- is - a lot to do with interpersonal relationships […] [This loneliness lasts] A couple of days or a couple of hours, a couple of moments only [laughs]… So, this differs a lot, depending on what [halt] currently simply happens, somehow.  I used to indeed feel lonely, also when I was somehow together with other people because - through different inner convictions and thoughts and so. Well, one kind of – so, that extremely covers that if one then doesn't just get oneself into that - into the other people, for instance, or thinks: “Yeah, I could now have, no clue - she or he is not really in tune with me and is it really of advantage for me now somehow to be with him or - with hi-him or h - uh with him or uh her - to spend time [halt]” - also in a group or something like that. “What's the point of that” or “where is it really?”, but - but that's just nonsense somehow. That's simply these thoughts - these convictions that one has - um, they can simply absolutely drag […] one down, exactly. |
| B1 (female, 44) | Prolonged loneliness (recovered); transient loneliness | Reports different situations of loneliness in her life but mainly short-lived or context-specific (e.g., after saying good-bye to friends; when with colleagues, who she does not connect with); says about herself that she does not feel lonely very often; reports one prolonged period of loneliness (seven years) | Interviewer: I will first ask you to tell me about a situation in, let's say, in the past year - when you have felt lonely.  B1: That would be hard. Well, maybe, for a moment, because, you see, I don't have a family but I have really great friends who I love very, very much and I see them as my family. And one of my best friends is 25-26 years old... and he went to study in the UK with his girlfriend, who is also a good friend of mine despite the age difference. We were inseparable and when they left, maybe for a moment, I've maybe felt lonely. It was not really something prolonged, it is more that the absence of some people makes you feel less good. But my other friends compensate for that absence, to some degree. Loneliness is more like a feeling of abandonment, like some sort of isolation, not having someone beside you when you need it. This rarely happens to me. I can’t think of other times… I lost all my relatives within a very short period of time. My mother, my father, all of my grandparents. Then was a time when I felt lonely and even scared from being all alone, and it is a slightly scary feeling to know that you are alone in the world and that you have to deal with everything alone. And again, thank God for my friends who didn’t leave me then and everything went back to normal.  Interviewer: And it [loneliness after saying goodbye to friends] continues for a longer period of time?  B1: No. Maybe for around a week every time.  […]  Interviewer: And when you lost your family, for how long did you feel lonely?  B1: Well… quite a while, maybe for a year.  This will probably not sound well but there are colleagues of mine with whom I feel very out of place. They don't understand you, you don't have anything to talk about when you're with them. […] Some people react strangely [to her unconventional ideas about teaching], and when I am in one place with more than two or three people that are like that, I don't feel quite well. Like alone, isolated, misunderstood. Only then. […] I have other people with whom I can team up with in situations like this. Usually, in other cases, if I feel like that, I am gone, I just leave.  I am a very curious person and maybe for that reason, I’ve rarely even felt bored or lonely because I always have something to do.  *Prolonged loneliness:*  I was very in love with one of my classmates - for many years, for seven years. I was so in love with him that I was feeling extremely alone even though I was surrounded by people, friends, but I wanted only him and nothing else in the world could replace his love. |
| B2 (male, 33) | Recurrent loneliness | Reports multiple different situations of loneliness throughout life, also in closer relationships, suggesting a personal susceptibility to feeling lonely | I can't think of a concrete example which had marked my consciousness. Of course, the periods and moments of loneliness come spontaneously, maybe due to interactions with others and other similar conditions. […] I would say that it is something that is systematic. It inevitably happens all the time, in specific situations. Even if I have to look back, there have been moments in my life when I have had more frequent periods of loneliness, but again, it was not 24/7 loneliness. Or, at least, it was not prolonged - even if it’s 24/7, it's due to something, an event. Something that made me feel lonely. Very rarely this will continue permanently, for a long time. After all, in everyday life you communicate with a lot of people, you have to get up every day and do stuff and that distracts you. […] Maybe, in most cases, the reason is really that it's due to communication with other people, due to life with others. […] I mean, one way or another, when you feel rejected, rejected from society, rejected from other people, that is when you feel lonely. You can feel misunderstood, or excluded from that particular society in many different ways and that is when loneliness occurs. |
| B3 (male, 33) | Transient loneliness | Reports loneliness as reaction to concrete situations or settings (e.g., work), but says that he generally does not feel lonely much | Over the past year, no. Because... this will be a longer answer but in general my job is very extroverted. My job includes meeting with people. I am introverted, even though I don't look like it. And actually, I feel better alone. So, the time I spend alone is pretty enjoyable because I "recharge" myself. I could think of older cases of loneliness but in the past one year, there were none.  But yes, I can highlight an example and it was actually in the past one year when I think about it. Until recently I was a manager of a company, an IT agency. And... Sometimes being a leader of an organization like this is a pretty lonely place. […] There are moments in which you feel "alone against the wind", like no one thinks in the same way as you. I mean you have one perspective, people in the team have another. There are plenty of moments in which a person says "Fuck, I'm alone here.", "It's pretty hard.", "I have no one to count on." So, even though I was not physically alone, the feeling was sometimes… very unpleasant, for sure.  I felt lonely, I've just remembered, a year ago. Maybe more, I don't remember. I found out that a person which I thought of as a close one to me - a friend, in a personal context, not professional, a close friend. And actually... I don't quite remember, the information came to me indirectly... That he actually doesn't want us to be that close anymore, that he doesn't want us to be on these terms anymore. We had this time in which we kind of drifted apart. I thought that it was just a phase because he was important to me. It turned out that it was a more a permanent thing for him. I didn't feel good for a few days, for sure, just because I don't have that many male close friends. […] I could call this a lonely period of time, from the perspective of male friendship. |
| B4 (female, 27) | Recurrent loneliness | Describes frequent loneliness experiences in everyday social situations, suggesting a personal susceptibility to feeling lonely | It's generally very specific in my case because my work involves working with many people, regular contact, and at the same time, loneliness is present as a feeling. […] As a choir conductor, I work with groups of people singing - whether it’s children or adults. This means constant work with many people, they're around you. But... You can feel very lonely in such a situation. Actually, the more unbearable thing in my opinion is being among many people and feeling alone inside because you can't trust them or - or because they have betrayed you, or because you feel that you have to behave in a certain way. This requirement to not be yourself also predisposes some kind of loneliness, in my opinion. Because you're not intrinsically close to these people, you just offer them what they expect. […] So, it’s kind of loneliness because you stay, um, y-you have to […] give other people what they want, but you cannot show yourself and your feelings and all of that.  For example, maybe this is actually appropriate for this conversation given the situation with my husband [they recently decided to live apart] because, um... There was a period when the two of us were together, but we felt lonelier. […]And also, that sometimes, you can be very close with someone but without uh – the - the physical distance doesn’t mean that that you are not – you’re lonely. And also, being close to someone physically doesn’t mean that you’re not lonely. |
| B5 (male, 31) | Transient loneliness | Reports that he hardly ever feels lonely | I don't think that I've felt that way - in many years, I haven't felt lonely. […] In the past five-six years, I haven't felt this way, at least not in a tangible way. […] Look, before that, there have been such moments. […] Well, I don't know, it's more, like, in relation to some type of rejection. For example, in a romantic sense, so to say. Um, I could have felt lonely in this sense. Otherwise, I've always been surrounded by people - just physically, friends and family included. Um, I haven't lived somewhere abroad for a while, for example, for there to have been a moment when I was all alone... and for that to have caused some loneliness - a feeling of loneliness [shrugs]. |
| B6 (female, 27) | Chronic loneliness (recovered), currently recurrent loneliness | Describes having felt lonely continuously from her childhood [when she did not understand it was loneliness yet] or puberty [supposedly more consciously] to young adulthood; through changing her social environment, she currently has recurrent but brief moments of loneliness only | If we're talking about a year or two ago, there was a long, serious period, when this thing was going on. But it wasn't only related to loneliness, but with depression, which I got treated. Like, I was actually getting it treated and this bad state lasted for about two years.  Before, it [loneliness experience] was a constant period, it didn’t end. That is, the moment I entered puberty and the moment I started being confused about which social group I want to be in - since then until around [the age of] twenty-something years, this loneliness was there. Like, when I was little, I didn't understand that this is typical loneliness. Then, when I started to understand, I started working on this much later. So, this loneliness really lasted years at the time, it didn't disappear. It was just years on end - an entire period [laughs], an entire period. Um... Now, when – now, when I have [these] periods... I said it - a week or two, it depends - to overcome this thing, to figure out what the problem is. […] But most of the time, loneliness, in my opinion, is provoked by many components and it cuts much deeper. But usually, I manage to deal with this thing within a few weeks until... the next problem comes along [laughs]. |
| B7 (female, 30) | Transient loneliness (intense loneliness) | Describes multiple situations of loneliness throughout life, but they seem to be bound to concrete external circumstances (e.g., intense loneliness when being geographically separated from close others for two years, stress at work, feeling distant from her husband for about a year) | Well, honestly, lonely in the sense of not being surrounded by people, not living with anyone, no. I haven't had those moments in the past year. But I have felt lonely, misunderstood, if loneliness could even be that... […] With me, it's more that my job is very demanding, very hard, very pressuring. And yes, I share that with the people close to me and they probably want to help me, but for now, they can't. That's more it. […] They listen to me, but, after all, they have their own lives, their own job. My parents, my husband - he also has a job. And no one can feel the type of empathy that, maybe, I wish someone can feel for me, because that person is not in my shoes. But... I wanted to tell you about the time I felt very lonely because I think that I can describe it very well. It was the time I was a student in Sofia. |
| B8 (female, 34) | Transient loneliness | Describes feeling lonely due to a few concrete external situations throughout her life (e.g., lacking help with household chores, moving for her studies, or a disharmonious holiday with friends) | I have to think about it. There definitely was [a moment of feeling lonely last year], but I don't remember exactly what it was. Something related to duties at home, things like that. Too many things piled up at once and I felt that I was doing everything by myself and no one was helping me. That was it. I can't think of anything else.  That was a very long time ago, when I left to study at university. That's when I was separated from family and friends for the first time, as [before] I had studied where I had lived, in high school, so… And at the beginning, I really felt very lonely, in a new place, without acquaintances. No acquaintances, no relatives - and you start everything from scratch. But, with hinsight, I guess, I've managed [laughs].  That was during a vacation at the seaside. I went with friends, but then… At the time, I really felt as though I was alone - both mentally and physically and so on. Because whatever we wanted to do, we couldn't, because each person had a different opinion, each person was pulling in different directions and overall, that was my worst vacation. |
| E1 (female, 28) | Recurrent loneliness (could also be viewed as transient loneliness) | Describes feeling lonely due to some concrete external situations (e.g., when working from home; due to not having a partner); as well as having felt lonely in situations that suggest a personal susceptibility to feeling lonely (e.g., when trying to make friends in the past because of not having learnt to make friends; if she has much work; if she’s not interacting with others at work) | There definitely is [a moment of loneliness in the past year], I just can't remember at the moment. […] There are times, for example, where I work a lot. So, during those busy work hours, even though I'm surrounded by people, I'm not able to receive help from them. […] So, I mean, perhaps the time when I felt most lonely was when I was working from home. So, when everyone in the house had gone out. Everyone went their separate ways and I was sitting there alone with the computer. […] Six months of only doing freelancing from home. […] And then, when I went to a new [working] place, uhhh, I was also still feeling loneliness, in a way. Because the team was different as well from the team that I had worked with for a while. [in English] So, let's say, maximum eight months.  [I also feel lonely] When those around me are busy with other things - rather than all of us working together on a shared topic. So, if I'm not pestering those around me and being pestered back by them, that's what makes me feel lonely.  Interviewer: Have you ever felt lonely due to the absence of a specific relationship in your life? For example, a partner or a close friend, and so on?  E1: A lot. For example, when I would transfer from one school to another school, I don’t have, uh, I’m n- I'm not able to make friends easily. Uh, so I used to have this problem, that- that I was homeschooled for a while. So, when I entered school, it was difficult for me to be - to form friendships. So, that led to me feeling lonely because I wasn't able to make friends.  That's the thing that could be, like, make me feel lonely now. To have someone who is a life partner - who shares everything with me then. |
| E2 (male, 33) | Transient loneliness (intense loneliness) | Describes loneliness due to concrete external situations, such as after his mother passed away and his engagement ended (intense loneliness); when with colleagues | The one time I felt lonely and socially isolated is when my mother passed away. For four or five months after her death, I was lonely and non-social. So, I was basically just working, I had no social life. I decided to get engaged, but it didn't work out. The whole thing lasted around a year and two months. […] Well, maybe I [felt lonely in particularly because I] didn't have a close friend who I could tell what's inside me. Most people around me were just work colleagues and these can't be told everything. […] What helped alleviating this feeling, is finding the partner I was looking for after the first relationship collapsed. I found someone whom I can speak freely with and who knows everything about me. This helped alleviating the feeling of loneliness. There is also another thing, which is building my own house and doing all the necessary preparations for marriage.  I feel like this [lonely] with colleagues who come from a different background and when there is a difference in the level of education. […] The problem I have with work colleagues is that my level of education is way better than theirs. So, when I try to chat with them, they get the feeling that I'm trying to show off, because I’m more informed. […] This leads to a certain feeling of loneliness where I truly think twice before engaging in a conversation with them. |
| E3 (male, 34) | Transient loneliness (intense loneliness) | Describes three months of intense loneliness after finishing school, when he could not follow the education he aspired and, relatedly, his romantic relationship ended; sometimes feels lonely at work | I did feel like that at a certain point of time. It was after school. After I finished school [in 2001]. Uh... I mean, it didn't last long. Because, back then, I was a bit upset... and then... because I took a path I didn't like in education. […] [For] Three months approximately. […] I was unemployed at that time. And I was out of school, so I had nothing to do. I was at home almost all the time. I mean, all the time at home. I didn’t go out or meet other people, or [did] anything. I wasn't even psychologically ready. I wasn't ready to interact with people. […] Coinciding with the period in which I felt lonely when I finished school, there has been a relationship... or, I had a relationship with a girl. She got married at that time. I mean... both things happened at the same time. […] and honestly, she broke up with me, despite the strong affection, she left me because I uh... she tried to discourage me from taking this educational path. And tried to show... tried to put me on the right track, so that I can win her heart in the end. But uh, it didn't work out because my financial situation back then wasn’t the best. So, she got married. […] And... at that time... losing her was one of the reasons why I felt lonely.  Well, due to my level of education, I was forced to work in a place I don't like. And on top of that, the company I work for has some dull-headed people... [laugh] Should I continue? It has people with mentalities far inferior to mine, intellectually speaking. [irrelevant] […] And when I do talk, it means that we'll chat about topics that are interesting to them, but not at all to me. I feel that way almost all the time when I'm at work. What makes up for it is being with my friends back home, like yourself. But at work, I do feel like that. |
| E4 (female, 25) | Prolonged loneliness | Reports having been feeling lonely for five years since losing her father; also reports having once felt lonely when a friend was not as close to her as she would have wanted; relatively little information available over loneliness pattern before her father’s death | E4: Uh... yes, when my Dad passed away. That was the time when I felt most lonely. […] There were close friends, but I still felt lonely. I feel lonely sometimes when I'm alone, and other times when I'm with others. I get both feelings. […] Since 2014.  Interviewer: So, all this time, you still have the same feeling?  E4: Yeah, definitely! […] Yes, this feeling is present. […] If it goes away, it happens for a very short period of time. But it's present most of the time.  She was my friend, so I wanted her by my side. To listen to me and I open up to her - and listen to her as well. Um... that we have general talks about something. I mean, not necessarily when I'm upset! On the contrary, I'd like us to always be together. |
| E5 (male, 44) | Prolonged loneliness (recovered); currently no loneliness | Felt lonely for 7-8 years about 20 years ago because of having different opinions and ideas than everyone around him; no other loneliness experiences | Over the past year and five years, there is none [no situation of loneliness]. The last time I felt lonely was... uh... like, twenty years ago. [laughs] Yeah. Back then, I wasn't married and... uh... in general… I had some intellectual differences with the people around me. So, I was always alone. Isolated myself in a room. [Because] Nobody understood me or what I was thinking of. And, in fact, they thought that I was headed somewhere else. […] You can say that... from... from high school uh... until the year... I finished high school in 1992, so up until... uh... 1999 or 2000. I was in total isolation. I was physically with them (people), but not mentally. Intellectual isolation. |
| E6 (male, 40) | Chronic loneliness (possibly recurrent loneliness) | Describes feeling lonely with financial issues – and feeling alone with them; although it is unclear how frequently he feels this loneliness, he reports taking different actions to escape his loneliness; also reports transient loneliness when working abroad in the past | Because there is no... perhaps at some point or time, I find out... there is no understanding. Or there is nothing particularly good, that makes you feel like coping with life. Suddenly, you may find yourself... as long as there is... hardships, things or problems, you feel like... hm... that's loneliness! […] I mean, if I have free time to get out of this feeling, in the sense that I... spend some time at a local cafe for instance. I go to... go visit a friend whom I haven't seen for a long time. Uh... I mean, anyone who is dear to me, I seek them out. You meet them to get out of this loneliness.  I went to Lybia... some time ago. I felt lonely at that time. […] No one of my family was with me. No friends, no one... This was really the time when I felt loneliness. […] [I stayed there for a] year and a half, maybe two years. But not one was with me, save God. [laughs] That's loneliness! One time you're... for example, with... with the family, and suddenly, you become... alone. |
| E7 (female, 35) | Chronic loneliness | Reports always feeling lonely, even when with friends (and despite good friendships), and makes a possible link with her childhood; also reports concrete situations of loneliness | I get that feeling [feeling lonely when with others] all the time. Although I know many people and have many good friends whom I love and who love me, I still get that feeling of loneliness even when they are around me, indeed. […] It could be attributed to things related to nurture, for example, or things I have missed since I was young, things I could not get. It could be attributed to the fact that I’m constantly looking for something I lack - this could be a person I’m looking for… You know, there are people that are always searching for someone that complements them - their other half. So, I feel this way. I feel that there is a lost part of me that I’m always searching for. […] So, yes, he will be the partner that I’m looking for, that I feel I’ve lost and that I’m looking for, but he will not just be the partner.  [There were] So many situations [of loneliness in the past year]. [in Arabic] Not only a specific one, uh, yes. […] I was living in a place and then, it happened that the owner of the place where I was living turned out to be a crook. And I had to leave this place where I lived and look for another one - immediately. So… So, at that time, I felt that I’m so confused and I wanted to ask the people around me for help, but I didn’t know how to do it because, as I said at the beginning, I don’t know how to ask for help. […] It took a lot of time! It took, maybe, like one month.  One of my best friends has got a scholarship abroad in the USA and she travelled there all of a sudden. So, I got kind of strong feelings of loneliness because she is - she is my best friend. |
| E8 (female, 26) | Recurrent loneliness (recovered); currently transient loneliness | Describes having felt lonely in groups in the past, but by now managing not to end up in prolonged situations of loneliness anymore (suggesting recurrent loneliness in the past); additionally desribes feeling lonely due to concrete external situations (suggesting transient loneliness now) | Yes [I sometimes feel lonely with other people], because I’m always with people who aren’t similar to me. They don’t think the way I think, they have another mentality. These people always tell me, “No, you think badly, you are wrong, you are….” So, I feel that I’m being alone, that my way of thinking is different from (the way) these people around me (think). So, I feel that I’m not in the right place, and that these aren’t the people I like to be with and talk to. […] I get this feeling only with a specific kind of people. But currently, I do not engage in any kind of relationship with people who aren’t similar to me. So, at this moment there are many people that are similar to me, and people who can understand what is going on in my mind or what I’m thinking about. These people are of course there!  Currently, I don’t get these feelings anymore as I did before, because I... I mean… I don’t want to say that I’m the only reason for this, but I can manage not to enter into a cycle that lets me have these feelings. For instance, if I think that I’m afraid or I have anxiety, and I feel that I don’t want to be alone, I will start to look for the people who are similar to me, who actually exist. I mean, I’m the one who starts to take the initiative and to contact (others). I’m a kind of person who - in the case that they need something from others - directly goes to them and asks them for it. So, no, I don’t feel lonely that much. When I feel that I’m alone, I can go into the direction of the person who can eliminate this feeling.  There was a person in my life, for some time - he was everything to me. He was my friends, my family, he was all these things in my life. So, when he started to pull back or withdraw from my life, I started to feel very lonely. […] I didn’t go out that much. I didn’t see and communicate with people that much. They [friends, family] tried to help me: "come on, let’s go out". They tried to be by my side, but I didn’t give anyone an opportunity to help. I didn't want that. There were some attempts, but they always failed. […] [It lasted] Between three and four months, approximately.  Uh, [another situation in which I was lonely was after] the death of my mother. It was a very big event in my life. She was my friend, my mother and my sister, she was everything to me. So, when that happened - sorry, I cannot go any further. I cannot explain that any further. |
| E9 (male, 25) | Transient loneliness (intense loneliness) | Has had clear loneliness experiences, but says about himself that he does not usually feel lonely much | Hm, most of the time, I don’t feel lonely. I’m not the kind of person who feels lonely that much.  I was engaged and after three years, I annulled my engagement. This is the period when I felt most lonely. […] [It lasted for] six months. […] No, I was always alone. Uh... no. Most of the time I was alone, even when I was… Even when there were people around, I always felt that I’m lonely. […]I wasn’t able to sit and have a chat with someone, to discuss with someone, to argue with someone. I was always by myself, in a dark room - I was totally alone. I just had my phone and my laptop in hand, only that. But it didn’t happen that someone talked to me or that we discussed something. But it was possible to have a casual conversation, like, “How are you?” – “I’m good, where are you going? What are you up to?” Just that kind of stuff.  Maybe currently, it could be that there are many people around me but I feel lonely. [inaudible] Because I just started with a new job, so I don’t know the people there. I was always with my friends in the old job, and I knew all the people there and we were very close to each other. But at the moment, I’m alone most of the time. |
| E10 (female, 25) | Transient loneliness (intense loneliness) | Describes an intense period of loneliness five or six years ago, due to not being close to her family or friends, and losing a potential partner; describes not feeling lonely since then | When I was young, I met someone uh... And, I mean, my family were somewhat distant from me - and I didn't have true close friends in my life. I needed someone by my side at that time. […] I was... in my second year in college - so, when…? 2014, so when I was around... 20 years old. […] For a year, there were no true friends around me, but afterwards, I met new people and new friends. They helped me not to feel that extreme version of loneliness I felt before - and that I'm not alone in life.  On the contrary, I didn't feel lonely at all last year. |
| I1 (female, 39) | Recurrent loneliness | Reports having felt lonely at multiple points of her life with an early onset, since she describes that her loneliness comes and goes in periods, her experience is categorized as recurrent, rather than chronic | I think that, in a general sense, throughout life, I sometimes feel lonely. And specifically, during this past year, I separated from someone that I was with, so I experienced particularly intense loneliness.  […] the period of my life that I lived in a city… I felt very detached. I felt that even when I was surrounded by friends and when I had meaningful relationships. Something about this urban experience, I think… like… it caused me to feel isolated, and it contributed to the period of loneliness. I think…  I think that the deep feelings of loneliness [emerged / came up] because of the relationships with both my father and mother. Um… I think that my connection with them inherently contributed to many periods of my life where I felt great loneliness. […] They [these feelings] began around the time of my parents’ divorce (a few years before the interview), and... they didn’t totally stop. They changed - they weren’t as hard as they used to be - but they still persisted. […] I think that it was mostly about a sense of security in the world – like, to feel – you know, that-that your parents have your back and that, if you – um, also that, if you need them, in a - in a serious crisis, that they’ll be there for you. But also, just - that they’ll sort of be there for you on a – on a more everyday basis. […]  And I’m not sure what is the chicken and what is the egg regarding this. Um... I can certainly say that... in the past, during these periods, when I felt a combination of all these things… when it was very extreme, I truly isolated myself and didn’t want to talk to anyone who came by because it was simply too overwhelming. Um... but in the periods when it was less extreme, I surrounded myself with many people in order to mask this feeling and distance myself from it. Um... and today... I think that I mainly try to befriend this feeling. […] my experience with Buddhist thought and practice really helped me to cope with this. Instead of relating to the loneliness as if it were attacking or harming me, or again, as if it reflected a personality flaw… I simply try, if I can - it is not easy - but to look at it and befriend it. And to remember that it will pass, because everything passes. And this really helps me.§  I think they [loneliness experiences] were - they were longer than moments. I think they were - like, periods of time. And then, they changed. Like - they didn't stay uh constant, but... […] I think um - I think it - when I was younger, it was harder for me to understand that that was what I was doing: It sort of took me time to figure out that I was repressing, you know, some of my wants and needs, so - sometimes it would come out - like, I - I would sort of uh - I would sort of have these outbursts of emotions that I wasn't even exactly expecting, because I held everything in too long and then um - I think that the more that I learned to express uh my feelings and - and my wants and needs in real time, so - uh - so that feeling just basically goes away. |
| I2 (female, 33) | Recurrent loneliness (could also be transient loneliness) | Reports loneliness in multiple situations of her life, where the situation itself does – for many people – not result in loneliness (e.g., travelling, being single for a short time), suggesting some susceptibility to loneliness | [I felt lonely] a little bit after he [son] was born. […] I think that it is something that every mom in our generation feels. I mean, in the western world at least. […] I assume that it’s also because it’s a first child and it is all new - and also because we have been living here for five years and I didn’t meet any people in those five years because I didn’t have the need for it. […] [It took] a couple of months, until I started to understand and also, until he grew older.  It happens to me a lot when I go abroad. When I go abroad, in general, it cuts me off my normal routine, casual daily life, and then, there suddenly is like a void, an emptiness.  I2: The loneliness came from the inside, the outside is not... like, it’s something that I have with myself - the difficulty of being alone with myself in peace. So, the environment is distracting, but it’s not solving the problem. […] I think that as a – like, most of my life, I was in relationships. So, I wouldn't be alone – so, I wouldn't have to deal with it. I assume that most of the people are like this. So, in periods between relationships… so, it's not that I really needed relationships… from my point of view, it was time to be with myself. But it was challenging, and then, those feelings came up. |
| I3 (male, 27) | Chronic loneliness | Participant says that loneliness has been escorting him for most of his life (after age 14/15/16); describes not fitting in with gender norms as main reason | […] it's hard for me to say... to define some kind of period, because I feel that the loneliness is… escorting me I would say for most of my life. Of course, there are points that it is more noticeable - I think that... I think that last year, there was a period of like a couple of months that I really felt alone in a really bad way. Like extraordinarily, and I also lived alone with no housemates.  I'm experiencing the sense of loneliness as a... as a feeling of absence, of, like, emptiness - like, there is a really big blank in my heart and usually, it goes to like… I mean, like, I only think about it in the context of… Yes, I miss… love, family, like, a close community that supports and hugs you.  I mean, around the age of 14-15, 16, you start designing your identity. Not just your sexual identity, also the religious one. And I was the “black sheep”. I think that, in all the programs and also at home, but also at college, in high school, and also in the youth organizations. And I just think that I felt really alone in the sense that I felt like I am the different one every time that people saw me as that different one and treated me like that. […]And all my life, I’ve been feeling it. |
| I4 (female, 26) | Chronic loneliness (possibly recovered) | Describes having felt lonely throughout most of her childhood and youth because of hiding that she was homosexual and because of distant family relationships; also describes intense loneliness in the previous year after a break-up and leaving the military | I think that I can describe it better when I compare it to my experience of being in the closet for many years. Both when I was in relationships, and when I wasn't - which was most of the time. The whole time, I felt like there was something separating me from my surroundings. Meaning, when this secret existed deep within me, burned me, and I couldn't share it, I felt like, even with my friends, I wasn't comfortable, and I felt lonely. Because there is something that prevents a truly honest conversation. Only after the breakup, I could speak honestly with people, without any barriers. Something about the experience of keeping a secret, and my internal tension - which I didn't give enough attention to - caused deep feelings of loneliness. Even at home, I lived a double life. At the Sabbath meal, I would say things, and they would have no connection to my experience.  I think that that my parents are the first people that come to mind because the truth is that it didn't make a difference whether or not I was in the closet. I always felt distant from my parents and I was fearful of what they would say and what they wouldn't say. And my mom is a very serious person. She is a kibbutznik. They don't talk about emotions. It's not an option. So, I think, this constant feeling that the atmosphere at home was not very maternal or supportive, and that I had to be incredibly independent. Actually, one of the reasons I considered staying in the army was because I needed a sense of security that was lacking at home. And the feeling that I have to figure out how my life is going to work out. Because that was the situation - that's my relationship with them. And this caused significant loneliness in my childhood. Now, with a psychologist, I can deal with it, and this is probably true with all parents - it's not like this is so unique.  If I were to summarize the past year, it would be a year at the center of which the word loneliness would be very present. I had a bad breakup around a year ago. […] In the beginning, I tried really hard not to be alone. I divided my time into minutes. And my psychologist emphasized to me not to be alone. I really focused on always scheduling meet-ups for myself - to the extent that I was obsessed with filling my schedule. |
| I5 (male, 37) | Transient loneliness | Describes mainly concrete external causes for feeling lonely, such as loneliness when moving places, when feeling like an outsider in primary school and at the military, as well as feeling lonely in significant life situations due to having lost his mother early on (these experiences may also suggest recurrent loneliness, but do not seem to occur very often) | Basically, it's really interesting because we've just been going through two years, where we moved from a very sociable place, that has a really great sense of belonging, communal - a place where everyone knows everyone, everybody knows us, everybody stops and greets you, has talks, chit-chatting - to a place where we really started over.  No, in elementary school. I remember when I was in elementary school. I remember very well that, in my final year of elementary school, I remember that, relatively, I felt that I was like… not entirely popular, but not entirely outcast. I can remember this experience very well - it was a really clear experience of me wanting to be part of – That I'm a part of a group, but that I don’t entirely fit in.  I remember experiences of loneliness during my service in the army, at the military base, when I was already starting my service - so, right at the active service. So, at the bootcamp, it was fine. And I remember that, during my time at the military base that I felt really lonely because I was really a 'dos' (religious Jew) within a military base that was filled with secular guys - and some gentiles, as well. It was really a feeling of loneliness - lots of really despicable guys, it was really a difficult service from this aspect, up until I found some normal guys to hang out with.  I think that I had more experiences of loneliness, now that I think about it… So, in recent years, I've been having experiences of loneliness when I suddenly feel the absence of my mother, as an orphan. […] Ever since I became a parent - that is, within the experience of parenthood, and within the experience of me suddenly getting my B.A, getting my M.B.A - all these key events that symbolize success in life, that's when I feel the loneliness. That, like, sometimes, I don't have my mother to share it with - it's unsolvable. |
| I6 (male, 35) | Transient loneliness | States that he generally does not feel lonely; describes a few situations of loneliness throughout life | So, specifically, in the past year, with respect to loneliness, I didn't feel it even for a second. […] I love being alone. A period of loneliness... It's something that I don't feel, even when I'm alone and even when I'm with people...  This first thing that comes to me is a long, long time ago. It was in the time when I was in Guatemala. I participated in a course that was extremely spiritual and profound. And at the end, it concluded with 40 days of silence. So, there was something related to loneliness that was very present and very substantial.  Yes, because again, nowadays, I never feel lonely. I never feel lonely. […] In my work - I was in a position in Greece - there was a period that was extremely difficult and stressful, where I felt that I was alone. Despite having partners, despite having a community around me that was supportive and invested in me - my father worked alongside me - I still felt alone. Because it was my responsibility to deal with things that were extremely difficult and complex. And I didn't have anyone to turn to. […] Two to three months of intense days that were stressful. |
| I7 (male, 31) | Prolonged loneliness (recovered), currently transient loneliness (could also be viewed as recurrent loneliness) | Describes having felt lonely throughout childhood until moving away from his family at age 14; repeated experiences of loneliness during his military service due to evaluation moments; and during his current studies because of being older than others | To be honest, [I have felt lonely] a lot. I think that I felt the sense of loneliness also in my childhood because of the situation in my family. Because my parents are divorced - they divorced when I was three years old. And my mom also has communication problems - she had a stroke and she can’t talk, she can only write. She doesn’t speak - she barely has any intonations, and they are very coarse. So, it’s really hard to understand things like feelings. So, because of those things I already felt loneliness in my childhood. […] from the age of 0 on, I remember my mom as someone who can’t speak. And I grew up with her - my dad didn’t raise us and had a new relationship with someone who divorced, too. And they married and have a boy, and she has three kids from the divorce, too. So, a lot of siblings, but still that sense of loneliness. […] But I can say that, when I decided to move to Haifa to the military boarding school [at the age of 14], it made me feel very relieved. For me, it was like a new start and I rebuilt my self-confidence and - like, I built my identity and, like, reinforced my self-confidence.  And also, in the air force course and the military, there is a lot of loneliness. Because there is a lot of egoism and there is some kind of lack of sharing feelings and emotions. […] In the period of the military, I think that it was very much like a “sinus” curve. [based on the regular evaluation moments in the military] […] For example, if you’re rated low or someone gets you off that course, you really feel like a failure and that makes you feel bad.  I don't think that I can, like, boil it down to a specific period but, just having the experience that two years ago, I started (university) studies in a mostly young environment - like, the average age is 25 and I'm 31 - is already giving me a sense of loneliness. |
| I8 (female, 32) | Recurrent loneliness (recovered); currently transient loneliness (intense) | Describes that she would feel lonely in various settings in the past, and still feels lonely when in contact with people from her past; reports strong reaction to external situation in recent past | I think that my sense of loneliness stopped mainly because of things that I changed in my mindset, which, as a result, brought more people around me. But it was first of all a change which comes from within and not a change - it's not the people from the outside. So, I think that this is more of how you feel with yourself… you feel… like, the world doesn't understand you… That you can't find some kind of purpose for yourself, some kind of goal, something that you do that is making you understand why you were put here on this earth. So, you'll feel lonely, no matter how many people you have around you […] It's not like I said, "Oh, alright, this is what you do to stop the loneliness. You should work on it." This is an understanding that came along the road, because in the beginning, you try many different things. And in the beginning, I filled my life little by little with lots of people who are partners in these experiences and share the same feelings. And nevertheless, you still continue coming back home with that weird feeling. […] And even when I was in relationships - long-term relationships. And it wasn't what made me feel different from what I'm feeling now. It truly comes down to life experience. […] I used to feel lonely very easily. I used to experience loneliness much more frequently - if we had been talking to each other a year and a half, two years ago... But today, it's very hard for me to even recall this feeling - like, how I was back then, really. I'm not saying that it won't return! In a year from now, I can (again) like my position less and notice that I'll again be in… I don't know if it's a cycle or not - I'm still too young to know that. It may be a cycle but I don't know it yet. So, at the moment, this matter of peace of mind is, like - of some kind of self-acceptance, is really true (that it helps).  I think that friends from the past can make me feel more lonely. […] Their presence, their presence in my life. And honestly, even though I love them very much… we now only meet once every few months. Um... I think that family also increases loneliness for me, sometimes.  The most powerful experience of loneliness that I felt was after the <FESTIVAL> last year, when I was obviously surrounded by lots of friends and it was very, very nice. […] the sense of loneliness after that - after a week of being surrounded by people - to suddenly be all by myself - was insanely powerful. So that I sat on the couch and cried for, like, two hours - with a duvet and ordered pizza. […] But, let's say, I think, more specifically, it was three or four days, where I didn't even go to work - like, for two days. When I was totally wrapped up in this sense of loneliness. |
| IN1 (female, 38) | Prolonged loneliness (recovered); recurrent loneliness in current life | Seems to have felt lonely for a long time in childhood due to a strict upbringing and introversion; describes herself that she felt lonely at many different points of her life because of being too introverted to share or because of not being suffiently supported | Uh, loneliness is like, not one year – like, you keep feeling that very often. I- I keep feeling that very often. I mean, not only now, might be even college days or childhood. I mean, there are so many incidences where I really felt lonely.  I'm little introvert kind. So, I don’t share anything much with people – so, that is when, like, I felt as though I cannot share. Even though there are people with whom I can share, but I’m scared because sometimes they might misuse information that you provide them or they might take advantage of what you share. So, I’m always scared. So, in those moments, when I desperately wanted to share something very important or I wanted an advice, because of my introvert or something, like, I always felt lonely. […] I mean, I cannot recall one situation. There are – in my life, there are n [?] number of situations. There are many, uh, might be like – might be just - take an example of a job. So, you are in like, you are in confused state whether you’ll be there in the job or there’s insecurity in your job. So those times, like, you cannot share with your friends. […] Or might be there are some financial problems also. So, in those cases also, you cannot share because you cannot ask your friends and all.  Like, if you're too depressed, if you're feeling very lonely, it might take at least a week for me. Otherwise, if it's a normal thing, like might be two days, three days. But maximum, a week. Week or sometimes, I also remember there was a time where I took almost two to three weeks.  Like, might be in childhood, I was feeling very lonely, but after marriage - like, I've been married for eleven years – so, after that, I didn't have any kind of, like, I was like happy with my life or something, but in between, there are so many circumstances happen, like, in the past two years, which made me really lonely. Because there was things where I left my job and then I was doing business, and then I went - underwent lot of loss, and there was nobody to support it. […] No no, during childhood days, I felt very lonely. Like, my mum was very strict, and I could not share, and I was like very introvert - I didn't have much friends. I had friends, but I was not talking to them openly. I only listened. […] Uuuh, I was not outspoken like that. So - but then, after marriage, also like, ok, like uh I started adjusting. |
| IN2 (female, 24) | Chronic loneliness | Decribes herself that she has been feeling lonely ever since she can remember and for various reasons; loneliness started with distant family relationships in her childhood | And yeah, I-I would say: for as long as I can remember. […] It's - it's sometimes constant, yes, I- I feel lonely around people. […] I do not like crowds, so I feel most lonely around crowds and I don't um…  They [family] don't understand me as much as they want to, and I don't understand them as much as I want to. And they're not in any position to change their perspective about a lot of things, and that causes a rift, with the way we communicate. And like, all this makes things worse. It - although I love them to death, it's just very hard to communicate. And that come- gets in the way. And, yeah, then there's obviously - I give up trying to talk to them and when I do give up trying to talk to them, I'm lonely. […] Um, like I said, I have a very difficult relationship with my family and they haven't been the most affectionate. Um, I've been the sort of black sheep in my family, my brother has been the high achiever. […] And um, they've been incredibly strict. It's a very authoritarian um household. And I wasn't given a lot of affection, physical affection and all of that. So, I find myself on most occasions feeling touch stuff - I uh [laughs] want to hold hands with my friends or lean my head on their shoulder. Small little um ... feelings that let me know that I'm not feeling - I'm not that alone and there's someone who cares about me. […] I mean, I've always looked for um, my father in teachers, I've looked for significant others in friends, I've always looked for something, and that was damaging ... because ultimately, as grim as this sounds, people let me down. [laughs]  I don't want to compare my problems with other people, but they don't seem to be that sad [laughs] half the time. If they are, then they don't show it, and I don't want to be the only sad person in the room. So ... it takes a lot of toll on you if you pretend all the time. And therefore, I can't be around people, I keep pretending.  And um, last year, I'm talking about 2019, so, um - I had a very bad break-up. [L: Ok.] And... it wasn't that bad, I thou- I thought that didn't affect me but because, before that, I started feeling this loneliness very badly, which caused me to break up and push people that I know away from me. […] I'm not saying uh... my-my boyfriend tried - ex! - he tried to be caring, but it wasn't the same way I wanted it. So, I tried putting this ideal image that I had in my head into everything that he did and it wasn't enough and - I realized I was battling with myself about what I wanted. So, yeah... lack of - I guess, I'm looking for atten-affection. Um, platonic affection. Needn't be romantic - I mean, a lot of people around me are looking for that romantic - oh my God, I want that love - No. It's just, I think, the fact that I feel touched out [?] a lot of times comes to play with me being lonely. |
| IN3 (male, 45) | Transient loneliness | Describes feelings of loneliness in specific external situations, such as when his wife and son travel to his wife’s family once a year; or a prolonged period of loneliness when he experienced loss in his business and others started distancing themselves from him | Actually, when my wife - my family - goes back to the native [to visit wife’s family] for some days during the holidays, I sometimes feel lonely. For a long time – like, for two months. They go for two months. During those times, I feel lonely. […] Every year during the holidays, when they go, I feel lonely.  Yes, apart from this, in the time when I had my own business actually - when I had a few hiccups in my business and people who were close to me started distancing themselves from me and didn't want to associate with me. […] I felt very lonely. […] For some years – like, one or one and a half years - I was feeling like that. |
| IN4 (female, 29) | Prolonged loneliness; transient loneliness (intense loneliness) | Participant describes having felt intensely lonely in the last year because of feeling unsupported by her husband and in-laws (who she lived with); describes prolonged loneliness after the death of her sister in-law (for three years) | Why - because, because I feel lonely during difficult times, as nobody will help. I am going through these difficulties alone. I am living as if I was alone, I feel. […] I mean, my husband was not helping, I can tell that. So, I had to do everything myself and be alone. He would not go to work even after loafing around for six months - and he would still be at home. Due to problems like these, I had thoughts of living alone instead of living together. […]It was too bad, this year. It is very bad. […] It is too bad. It is worse because of my husband's side.  [translator explains that ‘the husband’s side can also include his relatives] From my husband's side only, I have experienced a lot of difficulties. […] when I am very sad, I continue to think about it. But sometimes, after five minutes, I am back to normal. But then again, if something happens, I feel lonely.  When my sister-in-law passed away - she was very close to me. […] My older brother's wife. She was very close to me. Even my mother was not that close to me, she was like that for me. After she passed away, I felt that my life is lonely - loneliness. She used to be with me in everything. After she was no more, I felt that I am all alone. I am living alone, I felt. […] I think it has been more than five years. […] For roughly three years, we were all thinking about it. After that, once her daughter was a little older, whatever I felt towards my sister-in-law was shown towards her kid. |
| IN5 (female, 25) | Transient loneliness | Describes only one experience of loneliness (lasting about three months), in response to being separated from her friends | Yes, I have [felt lonely]. Since I started working in the factory. When I started working there, I had friends for two months. And after the third month, we got separated. […] First, we all became good friends, almost ten of us. And gradually, we started separating and moved to different places. And then, in the fourth or fifth month, I was sad because of that. But now, I have a few new friends, and now I am happy. […] Only during a time, like, when you suddenly have to get separated from your friends, I felt lonely. |
| IN6 (male, 34) | Recurrent loneliness | Describes having felt lonely on and off for the past 7-8 years, due to financial problems and ‘household problems’ (i.e., problems with relatives), but for relatively short periods of time | I have felt it [lonely] a lot of times [in the past year]. […] I mean, there will be a lot of problems at home – I won't be able to tell them to others. Even if I told others, I don't believe that they would be of help to me. That's why I feel lonely. […] [I have been feeling lonely] For around seven to eight years. [A paraphrases] I feel it now and then. […] I mean, when they come and ask (about how he is doing) once a month, I’m fine. But again, when they ask for money, it is a problem. Then, I feel lonely.  Because friends went away. On the one hand my friends, and on the other hand, my mother. My mother is away from me [“doora” – could be psychologically or physically]. That's why I feel lonely. […]  Interviewer: How long do you feel lonely?  IN6: For about 3-4 hours. |
| IN8 (male, 27) | Transient loneliness (could have felt recurrent loneliness in the past) | Describes not having felt lonely recently, but when he had a socially unaccepted romantic relationship and when families found out about it; mentions having felt lonely more before getting married | I used to go to work and I had a relationship with a girl there. I loved her. […] The problem was her family and my family got to know. There were fights between the families because of that. They (girl's family) reprimanded me. Because of that, I felt like going away - somewhere far off. […] Ten years ago. […] After they got to know (about the relationship), they got me married when I was about 20 years [two years after his relationship]. So, after I got married, that feeling just vanished like that. Then I had a family, so I became involved in that. […] Before my marriage, I felt lonely. After my marriage, I don't feel it anymore. I am close to my friend [who he also got close to after his marriage]. I am good, I am living happily.  Interviewer: Before getting married, how long did you feel lonely? The time (period)?  IN8: Time? I don't know the time (period)... I used to feel it for a day, from morning till evening. It would be on and off. |
| IN9 (male, 35) | Recurrent loneliness (recovered); currently transient loneliness | Describes having felt lonely repeatedly in the past; for instance because of wanting to share certain experiences or questions with others; when having existential questions or knowing little about something; also when not feeling connected to others; these loneliness experiences seem to have decreased over time and with getting married | So, if - if I achieve something or if I don't achieve something, if-if somebody rejects or somebody says no... Then only [I feel lonely]. Otherwise, generally no. […]  Sometimes, I wanna share. […] If I achieve something, if I do something in my professional life, if I... mmm... if some of my results get accepted, I want to be - I want to share it in a positive way, uh, but, when it doesn't go well, when, whatever I try, it's not working - in those sense, I do - I do wanna share it. […] It's something, which I have learned over the time - usually, it used to be a lot - like, anything wrong happen in your personal life, which keeps coming back and which is hurting, that is the time that you want to seek help or seek sharing, uh... but over the time, I um, I – I - I seek help less, a bit less.  Yes, so, so, uh, it's not just about uh sharing uh one thing. Sometimes, I want to understand what exactly happened in the world. Dynamics like uh why it's not working, why something is working. What is others' perspective? I might be having limitation in my thinking. Uh... so, in that sense, I want to understand. I want to - one, just to find the knowledge.  No one to share with, that's one thing. And, probably, I didn't even know how to handle certain things - I didn't - I was not capable enough, I was not exposed enough. I was, probably - those are again... But s- but then still, if there is somebody who can help you, like, even, uh being, uh having - Like, being with your wife, it helps a lot.  Mmm, yeah, sometimes, I get bored, being alone. [laughs] It may not have any event. But yeah, uh, yeah, that's all I can think of, yeah.  No, these days, they-they quickly vanish, but sometimes, it used to last for months.  It- it sometimes, it- there's just no reason. Sometimes, uh, sometimes, there is no reason. I can't pinpoint anything. Uh - it does feel like lonely, like, there's nothing, um, what I'm doing - some, I don't know, some existential questions kind of a thing, like why - [laughs] so, yeah...  Interviewer: So, have you ever felt lonely while you were together with other people?  IN9: Yeah. Yes. When I sometimes, I don't connect. When sometimes uh... how hard you try, their way of thinking is different from you. |
| IN10 (male, 28) | Recurrent loneliness (recovered); currently transient loneliness | Describes feeling lonely when he feels like he does not fit in with groups who he is different from (e.g., because he does not like to drink); reports having recurrently felt lonely at school; feeling lonely less frequently now | Uh, in the past year, not so much [I have not felt lonely so much]. Uh because uh, I got married recently - in the past three months. […]  Sometimes it'll mean people interaction [loneliness will come from interaction with people], um, sometimes, it would also mean that you're a misfit into the society that you are currently living in. I mostly fall into the misfit category. In the sense that I don't feel- feel well-connected with the people around me. […] Uh, for example, in office, this has been a consistent scenario. So, some of the - most of the people in office: they like to drink, they like to party. Um, you know, occasionally, I mean, not regularly, of course. […] I don't feel lonely anymore when I move away from that situations because I've developed my own world. Uh, for example, I love to code.  So, I've become more - I mean, I'm moving towards the extrovert uh - uh, nature. [L: I see.] So yeah, that's why it's reducing, a lot. But earlier, I wouldn't say, it was - it did not - even before a year, it did not uh last for a long time. It was in- loneliness feelings was in spikes. [L: Yeah.] Uh, but then again, the frequency of those spikes is reducing.  I definitely don't feel that I can connect better. Because I'm just not in that frequency range. Um, so... But I'm more - I've become more accepting towards myself. |
| T1 (male, 28) | Transient loneliness (intense loneliness) | Describes two longer-lasting experiences of loneliness due to external events; these seem to be relatively intense | Of course, I have [felt lonely]. So, I guess I can think of two [situations]. And there was also a period during the pandemic where I felt that loneliness and a kind of disconnection from people, like I was on my own. I can say that I felt that way for about a year. […] As the pandemic progressed and we got used to it, as we settled into a routine and returned to normalcy, that feeling of loneliness and disconnection decreased, I think.  And I can consider the 4-month period when I went to the Netherlands for my master's in 2018, and the 3-4 months after I returned, as a period when I felt lonely. It was about 7-8 months in total. […] Because even when I returned here, back to Turkey, after making the decision to leave the Netherlands. […] I felt like people perceived it as foolish to leave such a beautiful life there and come to a country like Turkey, which is considered a third-world country with a difficult crisis. And that pushed me a bit more into my shell. I have a friend closest to me, [name]. I felt that he understood me more. But other than him, I felt like no one I had contact with could understand me. […] So, when I look at that period, it feels like a kind of self-erasure in my life, like a black hole, the deepest point of loneliness. |
| T2 (female, 26) | Transient loneliness (intense loneliness) | Describes experiencing overwhelming loneliness at the moment (due to friends being far away; relationship issues) | I used to even enjoy the feeling of being alone in the past. But now, it's a feeling that overwhelms me. For example, even though there are a lot of people around, I feel like there aren't that many. I can't reach my friends whenever I want. [because they live in different places] The distance feels too large - like sitting at a table, not being able to see, touch, or feel the person. So, we can't talk or share things deeply. I think that our interactions remain superficial. Sometimes, I feel very lonely even within a relationship, especially in my romantic relationship. I feel very alone in that too. […] just last week, for instance, I experienced an overwhelming sense of loneliness. I didn't know what to do; I felt trapped here. So, I went to a park, sat there for quite a while, listened to some things, and so on. […] It's more of a momentary feeling, but sometimes, you know, how should I put it? Even in a social setting, I feel it. Not just when I'm alone. |
| T3 (male, 26) | Chronic loneliness | Describes feeling lonely in general, particularly through interaction with other people (with small breaks, such as a month break after five months of feeling lonely); feels even lonelier now due to geographical separation from friends | Even when I'm in a group of friends I feel a sense of loneliness. […] Currently, I'm [geographically] in a place that's far from the center, far from social interactions. So, I'm distant from my social relations. […] Well, the current situation is a big factor in this, but before this, what I experienced was mostly feeling isolated in a friend group. I experience this feeling in certain periods every year. I mean, the main reason for this is generally that I scrutinize people's behaviors too much and they seem artificial to me. Including my own. My behaviors towards others seem artificial. It's like playing a role, acting. […] It's not a persistent condition. But this, I think, might be due to situations where I force myself to go with the flow of life, situations where I exhaust myself. […] I use a multitasking approach there. For example, I play games while watching something on the side. I never leave my brain empty. […] I might feel lonely for about five months, and then I won't feel lonely for a month. It's like I'm gathering some energy there, expressing myself, and then I start feeling lonely again. |
| T4 (female, 26) | Prolonged loneliness | Describes that she has been feeling lonely since the Covid pandemic (interview conducted in spring 2023 – i.e., for three years); and currently feeling strongly lonely because of failed plans to get married | Actually, I feel somewhat lonely right now. I mean, I am not alone, but I feel lonely. It's a kind of loneliness that stems from not being understood. I feel like, after the pandemic, people no longer understand each other. In fact, I believe that society has started to become more isolated and introverted. […] Nobody has tolerance for anyone else, or people don't really connect with each other. Personally, I feel like I'm not understood, but I also realized that I don't make an effort to express [or explain] myself. I started to believe that it's a futile endeavor [she thinks that explaining herself to people would not make any difference (for her to feel understood by them), so expressing her thoughts would be a futile attempt]. And this is also the case in my environment.  Yes, about two months ago, I decided to get married, but it didn't work out. Of course, this had a strong influence on me. I distanced myself from people even more and wanted to withdraw into myself. Because in that situation, I experienced such intense emotions that right now, or at that time, I think about so many things and feel so many emotions that I don't open up or want to be understood. […] Actually, right now, as I said, yes, I have my family, they are there. I have very close friends, yes, they are there. I am in contact with them, but I feel incredibly lonely every day. Because I'm going through a process that no one else is going through. That's why they can't understand me, and I don't want to be understood either. I constantly feel lonely because of this, but it doesn't touch [bother] me right now. |
| T5 (female, 25) | Prolonged loneliness at the moment; recurrent loneliness throughout life | Describes having felt lonely for the last five years because of having difficulty finding close friends in a different city; also describes very often feeling lonely because of feeling like she does not fit in with others (e.g., at work; with friends who are at a different stage in life) | Because I grew up in a different city than the one I currently live in, for the past 5 years, I have experienced this feeling of loneliness occasionally due to being away from my friends or close circle. […] I mean, as I mentioned, the lack of many people in my close circle here, not being able to establish friendships with people who are similar to myself, and not being able to find those people, generally creates a feeling of loneliness. […]Well, I always feel this sense of loneliness, but I don't always feel extremely bad. […] However, in situations where I want to share something, I prefer to share it with people I already know and trust rather than going out and doing it [share experiences] with other people. The feeling of loneliness arises when I can't share it with them. Otherwise, in my daily life, when I'm doing my routine tasks and chores, I don't struggle too much or feel that thing very intensely.  First, in my work environment, I have a very different mindset from the people there. But I can’t think of a specific incident or topic at the moment. However, recently, for example, I have some friends whom I'm not very close to but occasionally chat with. When I'm with them in a group setting, there are times when I can't find the right words to contribute to the conversation. For instance, discussions about marriage, having children, or being in a serious committed relationship with someone, topics where I don't feel I am at that stage [of life] yet, and the conversations continue in that direction while I struggle to find my place. |
| T6 (female, 26) | Recurrent loneliness (could also be chronic loneliness) | Describes that she generally feels lonely, but that it comes out more strongly in certain situations; she also indicates that she often feels lonely for just an hour and then manages to ‘bury’ that feeling more again; this suggests either recurrent or chronic loneliness | I generally feel like that [lonely], I think. […] Well, I actually experienced it recently, but my mood was affected by something specific. It was due to an argument I had with my family. […] I felt like I wasn't understood.  Actually, I have that feeling in general, you know, I’ve been having it for a long time. When I can't explain my problems to someone, I conclude that nobody understands me, nobody loves me, and that makes me very lonely. Yes, I mostly experience this issue in relation to my family. I don't draw the same conclusion from a disagreement I have with someone outside of my family. […] I mean, I don't know, it's like a feeling that comes on strong and then slowly fades away. It's like it's always there inside me. When something bad happens, it comes out. Then, as I calm down and my nerves settle, maybe I bury that feeling a bit, and it feels lighter.  Interviewer: How long do you think the feeling of loneliness lasted when you had an argument with your family? Can you remember?  T6: I think it lasted for about an hour, I'm not sure. |
| T7 (female, 20) | Transient loneliness | Reports feeling lonely due to external events; for instance, when separated from friends; when not fitting in with others who are from outside her social circles | When I first started university, I missed my friends from Bursa. So, I felt somewhat lonely. […] I [still] occasionally feel it, but it is somewhat related to when I started.  I: How long does this feeling of loneliness generally last when it comes?  T7: It lasts until I engage in some social activity.  Well, not for right now, but it [feeling like not fitting in with others] happened last semester. It happens when I join a group of people in school, like a group of people who know each other. But I don't really know them well. […]  I: Does it also give you any negative emotions, like feeling sad or angry?  T7: Yes, I feel lonely and sad. |
| T8 (female, 19) | Transient loneliness | Can remember only one concrete situation of feeling lonely in the past five years, and this siutation did not last long | Well, sometimes when I can't express my problems to people and they don't understand me, I feel a sense of loneliness. […] I had a jury for school a few weeks ago, and I was both sick and stressed about meeting the deadline. I felt a bit bad because people didn't understand my problem during that period. […] It didn't last long, actually. It could be a few hours intermittently within a day, [manifested] as irritability. |
| T9 (female, 27) | Recurrent loneliness (little information available; could be recovered from chronic loneliness) | Describes that loneliness started in childhood; describes concrete external situations causing loneliness recently (e.g., lacking opportunity to meet with others during the covid pandemic; not being offered a place to stay by her friends); although she does not make an explicit link with loneliness, she also describes not fitting in at school and feeling different at university because of having a stricter father than others, which might explain why her loneliness dates back to childhood; describes that she doesn’t feel the negative consequences of not fitting in anymore since she got married | It goes back to my childhood, to be honest.  When I was younger, during elementary and middle school, I spent 8 years in the same school, and I truly felt different in that environment. Socially, I felt different from the social status in the class, the atmosphere, and the profiles of the students. I thought I couldn't fit in with them. […] It could have been my family background, financial situation, or my family’s education and perspective.  My family isn't extremely conservative, but my father is a controlling person who presents his controlling behavior as [religious] conservatism. […] I was 18 years old, and I wanted to join the university club and so on. Rehearsing every Tuesday was like a battle [with the family] for me. When I came home late in the evening, I would always end up arguing. And eventually, when friends would plan to go somewhere, I couldn't go - I would delay it or make excuses. After a while, when you don't go so many times, you stop getting invited. So, when I couldn't go and wasn't invited to their gatherings anymore, I realized that I couldn't be part of that group. That's how I felt at the time.  I couldn't find a place to stay for those two weeks, and the friends I felt very close to - the ones I used to hang out with all the time - didn't open their doors for me [let her stay over]. I felt very lonely at that time. I mean, I would have definitely taken in someone in a similar situation. Of course, they all had their reasons. It isn’t like I blame them. When they explained, it made sense, but when I asked 3, 4, 5, 6, 7 people and received rejection, it became a bit frustrating. It felt wrong in this way, and then I went to stay with a friend with whom I didn't have a great relationship. She opened her house, and I stayed with her for a while, a few days. […] It lasted a long time - that feeling didn't go away easily. It felt like a void. […] As I mentioned before, meeting my friends regularly, spending time with them at the cafeteria, and so on, helped calm down that feeling of loneliness a bit. But deep down, something remained. |
| T10 (male, 26) | Transient loneliness | Describes only feelings of loneliness due to a specific situation (worries because of dog’s illness) | Well, I didn't feel completely alone, to be honest. I don't remember experiencing such a thing recently. There was just a period where I felt generally lonely, you know – just a general feeling of loneliness. Maybe from November last year until January of this year. […] My dog got cancer, and we were dealing with his treatment. […] Just when his condition was deteriorating, my mother fell down the stairs at the hospital and sprained her ankle, so she couldn't walk for a while. It was a period of running around like crazy. I didn't really explain my situation to anyone. […] I also felt that no one could do anything. Because the people I shared this with would ultimately ask if there was anything they could do, or they said “if I can help you with anything, let them know”. But there was not anything to do. |
| T11 (female, 25) | Chronic loneliness | Describes that she always feels lonely and cannot remember when these feelings started | Well, in general, I always feel lonely. It's like, you know, events, the flow of life, relationships - maybe the intensity within three days puts a curtain in front of this feeling, but at the end of the day, it's as if nothing can take away that loneliness. I always feel like I'm left to myself. I always feel like I'm left to myself. It's like, you know, it happens sometimes when you come home after a busy day, close the door, and go, "Oh, okay, the noise is over." But it's not like the noise is over; it feels like... Okay, we entered the house, we're left to ourselves again, I'm left to myself. I don't even remember when this started. It has been something that has existed within me for a long time as a feeling. Sometimes I forget about it due to busyness and stuff, but at the end of the day, this feeling always continues inside me.  Well, there was a moment a few months ago, at the beginning of January, when one of my closest friends had an epileptic seizure. […] It felt like everyone in the world had their own things going on, dealing with something, and I was the only one standing alone in the hospital emergency room with a lot of questions in my mind. […] It didn't occur to me to call anyone at that moment. There was a lack of both emotional and physical presence.  Of course, the past five years include the pandemic. During the pandemic, especially, it was a period of being in my room with my noise-cancelling headphones, just working while the outside world's noise was blocked. I felt like I was staring at empty walls a lot during that time, as if life was happening somewhere else and I was just confined within those four walls. |
