# Supplementary material for: A Moment Versus a Lifetime: Patterns of Loneliness and Perceived Causes in People's Lived Experiences
Source: Ann N Y Acad Sci. 2025 Oct 3;1553(1):172–86. doi: 10.1111/nyas.70082 (PMC12645267; doi:10.1111/nyas.70082)
Supplement: Supplementary file 2 — Supplementary Table: nyas70082‐sup‐0002‐tableS2.docx [file NYAS-1553-172-s001.docx]

**Table S2** Additional Quotes

| **Theme** | **Example quotes** |
| --- | --- |
| Chronic loneliness (additional example) | I feel that loneliness is… escorting me, I would say, for most of my life. (I3, male, 27, Israeli)  Well, in general, I always feel lonely. […] at the end of the day, it's as if nothing can take away that loneliness. I always feel like I'm left to myself. I always feel like I'm left to myself. […] I don't even remember when this started. It has been something that has existed within me for a long time as a feeling. (T11, female, 25, Turkish, chronic loneliness) |
| Personal susceptibility to feeling lonely among participants with recurrent loneliness | I used to indeed feel lonely, also when I was somehow together with other people because - through - different inner convictions and thoughts and so. (A9, female, 26, Austrian, recurrent loneliness) |
| Lack of affection and closeness due to unfulfilling family relationships among participants with chronic loneliness | First, that I didn't have a good relationship with my father. […] Never being able to talk to him, the same thing with my mother – like, my mother and I were close as mother and daughter, but we never talked about the things that worry me or excite me. Whereas, since I was little, I wanted to talk to her about these things - like, "[This thing] interested me, I want to share with you what's going on with me,", but there wasn't anyone back then to listen to me. […] Then I realized that I have to look for people around me with whom to share these things. And so, I look and I look, and in the end, you don't have anyone in your family to share it with, you don't have anyone outside to share it with and in the end, you, like, keep searching, searching and searching. (B6, female, 27, Bulgarian, chronic loneliness - recovered) |
| Not fitting in among participant with prolonged or recurrent loneliness | I had some intellectual differences with the people around me. […] Intellectual isolation. […] For example, they suggested that I should learn to drive a car. At that time, I was a bit idealistic. So, I was thinking: how would I drive a car and be responsible for the impact of its emissions? Back then, nobody understood a word of what I was saying. They thought that either I was mad or I had some mental issues. (E5, male, 44, Egyptian, prolonged loneliness)  So, some of the - most of the people in office: they like to drink, they like to party. Um, you know, occasionally, I mean, not regularly, of course. Uh, but I'm not that kind. So, I - I don't like to drink, I don't like to party. I don't fall in that, you know, uh that category of people at all. So, let's say, but, in some instances, I cannot avoid going to a party. For example, there's gonna be a promotion party, uh there's gonna be a- a celebration party for product development. Something like that. So, if we head out over there, then I'll usually end up sitting in a corner, and having uh - having Sprite or Coke or something - anything - pizza, whatever - by myself. So, I feel very lonely at that point - at that point in time. So, basically, it's the- it's the misfit in the environment that I'm uh - there. (IN10, male, 28, Indian, recurrent loneliness) |
| Not fitting in with others among participants with transient loneliness | Or, now I remember, if I think about it - with - with friends or acquaintances who still like to drink at my age and if one, for example, doesn't join for drinking that much, then it can, for example, happen that, on that evening [halt], one has a slight loneliness, simply because - because you don't belong at that moment [ja?]. And then you are not in that mood and these are such moments of daily life [ja?] that I wouldn't - uh - attach too much importance to now, but - but yes, that - that exists after all, yeah. […] Or, I've been on holiday with a group like that last - last winter, where really everyone permanently - well, skiing vacation, ja? - and I like skiing - um, but among them, it's more about drinking than about skiing and something like that is not really my thing. […] I also don't have such a problem to say "ok, you [others] go drink - I just ski and I maybe join once and otherwise I just stay at the appartment, ja? That is also not that dramatic for me, but of course, you are then out [of the group] a little, ja? (A7, male, 28, Austrian, transient loneliness)  Like, the moment you feel out of place, maybe, or something is missing, um... […] It's been rather rare, and for example, because [at some moment] I wasn't with my own friends - for example, around other people that I'm not that close to. Like, if I ended up somewhere where I was out of place or I didn't feel... But it's still occasional, and not... It didn't happen regularly and it wasn't planned all that much, hmm... For example, if I went somewhere on a school trip as a kid, maybe I didn't feel like going and so, I felt lonely. (B5, male, 31, Bulgarian, transient loneliness)  The problem I have with work colleagues is that my level of education is way better than theirs. So, when I try to chat with them, they get the feeling that I'm trying to show off, because I’m more informed. So, they think that I try to pretend to be a wise guy. The reality is, when we chat and I have a certain fact or info about a topic, I feel the urge to drop it to negate their nonsense. This whole thing is a result of their low level of education. This leads to a certain feeling of loneliness where I truly think twice before engaging in a conversation with them. (E2, male, 33, Egyptian, transient loneliness) |
| Systematically not fitting in with others among people with chronic loneliness (additional example) | I was - for instance, during primary school, […] I felt very lonely there because I was a foreigner in - in - and one made me feel that. I don't believe that the children did this on purpose, but society does that, and the children hence do that, too. (A1, male, 35, Austrian, chronic loneliness - recovered) |
| Sensitivity, hypervigilance, rumination, and overgeneralizations in relationships among participants with transient loneliness | Feeling alone sometimes makes you close yourself off and sometimes you don't communicate properly with your loved ones. You think that no one understands you... Maybe that's not entirely the case, but that's the perspective. (B3, male, 33, Bulgarian, transient loneliness)  Like, the moment you feel out of place, maybe, or something is missing, um... In a sense, there have been such periods of time, but they were more so purely emotional - like, they were not provoked by some event, maybe. (B5, male, 31, Bulgarian, transient loneliness) |
| Rumination about relationships by participant with currently intense loneliness | But like I said, I perceive everything as a threat to myself, as if everything was about me. My thoughts, what's happening around me, how people behave towards me, everything feels very important. That's why my brain is constantly in a state of control, analysis, always like that. ‘What is this person feeling towards me right now? How did they do this or that? How did they put this or that? It seems like they don't want me anymore - okay, I shouldn't be with this person.’ For instance, ‘I can’t do something with this friend - okay, it shouldn't be.’ I’m constantly analyzing. (T2, female, 26, Turkish, intense current loneliness) |
| Conflict or relational issues among people with recurrent loneliness | With us, well, with me, privately, it has to do with parenting. That is, [partner] and I have very different parenting styles and, of course, it's up to [partner] to educate the children - they are her children after all - um - and there - that then also sometimes creates loneliness. […] because Tina [partner] in that case then takes a different decision than I would have taken and that then often leads to conflict and - I then withdraw because of that, and that then kind of creates an inner loneliness. And of course, I would in that moment kind of wish for a girlfriend who parents in the same way as I do or, kind of, children who maybe don't get you in these situations, ja? (A8, female, 35, Austrian, recurrent loneliness) |
| Unfulfilled relationship expectations among participants with transient loneliness | Yes, and it was maybe rather related to certain people with whom I wanted to have some kind of relationship - whether a friendship or something else. That is, again, in the sense of not "alone" in itself, but more like a certain thing that you want, but you don't have. […] in a sense I was looking for a type of company, "soulmate", like - I don't know the word in Bulgarian. Or something similar in that sense. (B5, male, 31, Bulgarian, transient loneliness)  But for the close friend, I maybe had very high expectations and was eventually let down. But not by all of them (friends). (E10, male, 25, Egyptian, transient loneliness) |
| Discomfort with oneself (additional examples) | I very much felt this feeling of loneliness [after her break-up] even in simple things. Suddenly, deliberations that I never had, such as, “what do I want to eat?” - suddenly, I found myself thinking about this. Like about a critical question about my identity. In reality, it's a simple question: do you prefer this or this? And for me, it appeared to be an essential question about who I am. And these were instances that kept repeating over the course of the year: to examine who I am in all of these things. (I4, female, 26, Israeli, chronic loneliness)  The loneliness came from the inside, the outside is not... like, it’s something that I have with myself - the difficulty of being alone with myself in peace. So, the environment is distracting, but it’s not solving the problem. I think that as a – like, most of my life, I was in relationships. So, I wouldn't be alone – so, I wouldn't have to deal with it. I assume that most of the people are like this. So, in periods between relationships… so, it's not that I really needed relationships… from my point of view, it was time to be with myself. But it was challenging, and then, those feelings came up. (I2, female, 32, recurrent loneliness) |
| Recovered from recurrent loneliness through more self-acceptance | I think that it [feeling less lonely] is the result of my relationship with myself. Like, if I'm in a good and constructive relationship with myself - like, this was the change that happened to me - so, also the relationships that I'm building as a result of that, are more real and strong and honest. And when you don't have this connection with yourself, then your surrounding is less communicative with you. (I8, female, 32, Israeli, recurrent loneliness - recovered) |
| Recovered from chronic loneliness through coming out of the closet | The whole time, I felt like there was something separating me from my surroundings. Meaning, when this secret existed deep within me, burned me, and I couldn't share it, I felt like, even with my friends, I wasn't comfortable, and I felt lonely. Because there is something that prevents a truly honest conversation. [Once I came out of the closet] I could speak honestly with people, without any barriers. Something about keeping a secret, and my internal tension - which I didn't give enough attention to - caused deep feelings of loneliness. Even at home, I lived a double life. At the Sabbath meal, I would say things, and they would have no connection to my experience. (I4, female, 26, Israeli, chronic loneliness - recovered) |
| Recovered from chronic loneliness through getting more comfortable spending time alone | This is what I am discovering right now. That I withdraw and don't feel lonely. […] But earlier, it was different for me. Exactly, that withdrawal is loneliness. And I actually wanted to get among people more, you know? But somehow they did not accept me really. […] I also need solitude. To understand myself, to find myself. To walk that - you know, spiritual path [halt]. And there, I have noticed that solitude is good for me, and there I find myself and I can - meditation and, yeah, zen. (A1, male, 41, Austrian, chronic loneliness) |
| Recovered from chronic/recurrent/prolonged loneliness through getting married | If there is somebody who can help you, like, even, uh being, uh having - like, being with your wife, it helps a lot. No, these days, they-they [moments of loneliness] quickly vanish, but sometimes [before getting married], it used to last for months. (IN10, 28, male, Indian) |
| Recovered from chronic loneliness because of becoming a father | The thing is that, for one and a half years, I have been having the luck to have a daughter and that fills out my life quite a bit. And - and I have to say that, since then, I feel least lonely. […] Between 16 - 16 and 20, I started to somehow create my identity anew and to have new friends […] and then maybe, for the first time, I felt less lonely. But I’ve only been feeling really not lonely anymore for the past three or four years. And really, really not lonely for 1,5 years. (A1, male, 35, Austrian, chronic loneliness - recovered) |
